# Integrative Single Cell Atlas Revealed Intratumoral Heterogeneity Generation from an Adaptive Epigenetic Cell State in Human Bladder Urothelial Carcinoma

**DOI:** 10.1002/advs.202308438

**Published:** 2024-04-06

**Authors:** Yu Xiao, Wan Jin, Kaiyu Qian, Lingao Ju, Gang Wang, Kai Wu, Rui Cao, Luyuan Chang, Zilin Xu, Jun Luo, Liuying Shan, Fang Yu, Xintong Chen, Dongmei Liu, Hong Cao, Yejinpeng Wang, Xinyue Cao, Wei Zhou, Diansheng Cui, Ye Tian, Chundong Ji, Yongwen Luo, Xin Hong, Fangjin Chen, Minsheng Peng, Yi Zhang, Xinghuan Wang

**Affiliations:** ^1^ Department of Urology, Hubei Key Laboratory of Urological Diseases, Department of Biological Repositories, Human Genetic Resources Preservation Center of Hubei Province Zhongnan Hospital of Wuhan University Wuhan 430071 China; ^2^ Euler Technology Beijing 102206 China; ^3^ Medical Research Institute Wuhan University Wuhan 430071 China; ^4^ Department of Urology Beijing Friendship Hospital Capital Medical University Beijing 100050 China; ^5^ Department of Pathology Zhongnan Hospital of Wuhan University Wuhan 430071 China; ^6^ Clinical Trial Center Zhongnan Hospital of Wuhan University Wuhan 430071 China; ^7^ Hubei Key Laboratory of Medical Technology on Transplantation Institute of Hepatobiliary Diseases of Wuhan University, Transplant Center of Wuhan University Wuhan 430071 China; ^8^ Department of Urology Hubei Cancer Hospital Wuhan 430079 China; ^9^ Department of Urology The Affiliated Hospital of Panzhihua University Panzhihua 617099 China; ^10^ Department of Urology Peking University International Hospital Beijing 102206 China; ^11^ Center for Quantitative Biology School of Life Sciences Peking University Beijing 100091 China; ^12^ State Key Laboratory of Genetic Resources and Evolution Kunming Institute of Zoology Chinese Academy of Sciences Kunming 650201 China; ^13^ Kunming College of Life Science University of Academy of Sciences Kunming 650201 China

**Keywords:** adaptive immunity, bladder cancer, intratumor heterogeneity, single cell, TM4SF1‐positive cancer subpopulation

## Abstract

Intratumor heterogeneity (ITH) of bladder cancer (BLCA) contributes to therapy resistance and immune evasion affecting clinical prognosis. The molecular and cellular mechanisms contributing to BLCA ITH generation remain elusive. It is found that a TM4SF1‐positive cancer subpopulation (TPCS) can generate ITH in BLCA, evidenced by integrative single cell atlas analysis. Extensive profiling of the epigenome and transcriptome of all stages of BLCA revealed their evolutionary trajectories. Distinct ancestor cells gave rise to low‐grade noninvasive and high‐grade invasive BLCA. Epigenome reprograming led to transcriptional heterogeneity in BLCA. During early oncogenesis, epithelial‐to‐mesenchymal transition generated TPCS. TPCS has stem‐cell‐like properties and exhibited transcriptional plasticity, priming the development of transcriptionally heterogeneous descendent cell lineages. Moreover, TPCS prevalence in tumor is associated with advanced stage cancer and poor prognosis. The results of this study suggested that bladder cancer interacts with its environment by acquiring a stem cell‐like epigenomic landscape, which might generate ITH without additional genetic diversification.

## Graphical abstract

A subpopulation of bladder cancer cells, characterized by TM4SF1 expression, is identified as a source of intratumor heterogeneity (ITH). This group shows stem‐cell traits and promotes diverse cell lineages within the tumor. Their presence correlates with advanced cancer stages and worse outcomes, suggesting they drive ITH through epigenomic reprogramming rather than genetic changes.

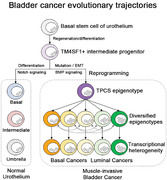

## 1 Introduction

Globally, bladder urothelial carcinoma (BLCA), a complex and heterogeneous disease, causes 212 500 deaths each year.^[^ ^1^ ^]^ The biological behavior of BLCA is determined by its phenotypic diversity or intratumor heterogeneity (ITH). ITH is correlated with clinical stages and less favorable survival outcomes in BLCA^[^ ^2^ ^]^ and other cancer types.^[^ ^3^ ^]^ Previous studies on other cancer types have suggested that epigenetic plasticity^[^ ^4^ ^]^ and genomic mutations^[^ ^4^, ^5^ ^]^ contribute to the development of ITH. However, the precise molecular mechanism involved in generating ITH in BLCA has not been elucidated.

Clinically, BLCA is classified into the following two distinct types: the less malignant non‐muscle‐invasive bladder cancer (NMIBC) and the aggressive muscle‐invasive bladder cancer (MIBC).^[^ ^5^, ^6^ ^]^ Pathological grading classifies BLCA into papillary urothelial neoplasm of low malignant potential, low‐grade, non‐invasive, and high‐grade, invasive cancers.^[^ ^5^ ^]^ According to a proposed molecular classification system, MIBC is further divided into luminal and basal categories.^[^ ^7^ ^]^ Animal studies have elucidated the different cell‐of‐origins of papillary and basal‐type high‐grade invasive cancers,^[^ ^8^ ^]^ which resemble human papillary, low‐grade BLCA or invasive, high‐grade BLCA, respectively.^[^ ^9^ ^]^

Recent studies have revealed the presence of stem‐cell‐like subpopulations during oncogenesis in various cancer types,^[^ ^10^ ^]^ including BLCA. These cells can differentiate and give rise to heterogeneous progeny, contributing to ITH. In a rodent model of BLCA, tumor‐initiating cells with basal‐like and epithelial‐to‐mesenchymal transition (EMT)‐like gene expression signatures can differentiate to adopt multiple lineage markers. Similarly, in patient‐derived xenograft models of MIBC, isolated human CD49f^low^ MIBCs give rises to both CD49f^low^ and CD49f^high^ progenies, indicating lineage plasticity in human BLCA. However, a specific molecular marker for these CD49f^low^ tumor‐initiating cells has not been identified.

Here, we analyzed genomic mutations, epigenetic modifications, and RNA expression in human and mouse BLCA at a single cell (sc) resolution to evaluate tumorigenesis and ITH generation in BLCA. The results of this study demonstrated that the basal cells of the normal urothelium give rises to MIBC. In MIBC, a TM4SF1‐positive cancer subpopulation (TPCS) contributed to ITH. TPCS, which arises during early BLCA evolution, is selected by immune cells during cancer progression. Functionally, TPCS have plastic transcriptome that does not require additional genetic mutations to support adaptive phenotypic shifts. This study revealed a comprehensive natural history of BLCA evolution to identify the key molecular events and cellular factors mediating the pathogenesis of this disease.

## 2 Results

### 2.1 Evaluation of BLCA Evolution at a Single Cell Resolution

**Figure 1:**
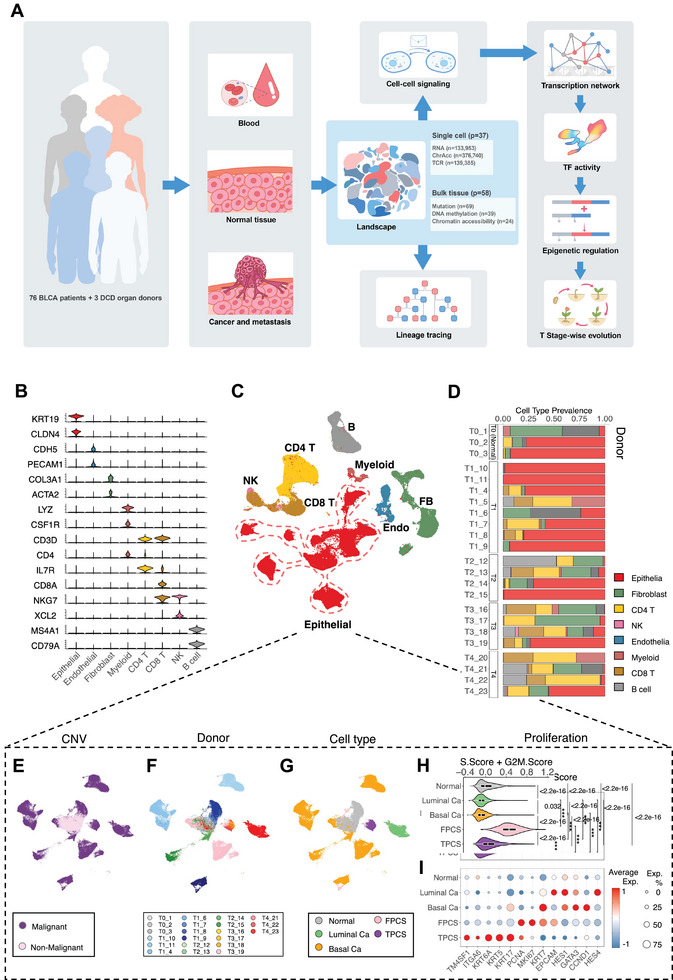
Single cell atlas of BLCA. A) Schematic representation of experimental strategy for the study. To understand the tumor evolution during bladder cancer progression across clinical stages and therapy, a multi‐center human study was performed. A total of 79 donors were included in the study: 65 patients with BLCA patients and 3 DCD controls were recruited from 4 centers, alongside with 11 BLCA patients from a public scRNA dataset (PRJNA662018). Genomic mutation, DNA methylation, chromatin accessibility, RNA expression and TCR rearrangement were profiled for bulk tissue or single cells. Bioinformatic analysis including cell‐cell signaling analysis, transcription network construction, transcription factor activity measurement and epigenetic regulation analysis were subsequently performed. B) Marker gene expression for each cell type. C) Uniform manifold approximation and projection (UMAP) of scRNA sequencing data from bladder tissues. Epithelial cells, fibroblast (FB), endothelial cells (Endo), macrophages and monocytes (Myeloid), CD4^+^ helper T cells (CD4 T), CD8^+^ cytotoxic T cells (CD8 T), natural killer cells (NK), and B lymphoid cells B) were shown. D) Cell type compositions from each patient/donor. Cell type was colored same with C). Donor/patient ID on Y axis was designed by combing the tumor stage of a donor with a sequential identification number. E) Based on the copy number variation (CNV) in scRNA‐seq data of epithelial cells, cells had classified them into malignant (purple, with CNV) and non‐malignant (pink, without CNV) populations. F) Donor contribution mapped on the UMAP of scRNA‐seq data of epithelial cells, indicating that donor‐specific transcriptional heterogeneity dominated the differences between these cancer cells. G) Major cell type classification of epithelial cells. H) Cell cycle‐related gene expression profile (S.Score + G2M.Score) of each major epithelial cell population. I) Differential expression of selected genes between the major epithelial cell populations. *P*‐value: Wilcoxon test.

To investigate the multifaceted nature of BLCA, encompassing genomic mutations, DNA methylation, chromatin accessibility, and RNA expression, 79 donors were included in the study. This cohort comprised 65 BLCA patients and 3 individuals who donated after cardiac death (DCD) directly involved in our research, alongside 11 BLCA patients from the publicly accessible dataset PRJNA662018 (**Figure** **1**A; Figures S1 and S2, Supporting Information Dataset 1). We conducted genomic mutation profiling on tumor samples to identify copy number variations (CNV, Supporting Information Dataset 2) and short nucleotide variations (SNV, Supporting Information Dataset 3).

To characterize the evolution of BLCA at the single‐cell level, we combined single‐cell RNA sequencing (scRNA‐seq), single‐cell T cell receptor sequencing (scTCR‐seq), and single‐cell assay for transposase‐accessible chromatin sequencing (scATAC‐seq). These sequencing and analysis were performed on 56 tissue samples from 26 donors, encompassing 23 BLCA patients and 3 DCD donors (Figure 1A; Figure S1, Supporting Information Dataset 1). Furthermore, we enriched the scRNA analysis by integrating endothelial and epithelial cells of 11 donors sourced from PRJNA662018 dataset (Figure 1A; Figure S1, Supporting Information Dataset 1).

Obtaining truly non‐invasive, low‐grade BLCA samples suitable for single‐cell sequencing is improbable owing to the clinical protocol. Of the 23 patients subjected to sequencing analysis (Figure S1, Supporting Information Dataset 1), only one was histologically classified as having low‐grade Ta BLCA. Furthermore, the somatic mutation profile in this patient was atypical for Ta grade, as *FGFR*/*TERT* mutations, which are typically associated with Ta, were absent in this tumor. All cancer cells in this patient belonged to the basal subtype. Meanwhile, all luminal cancer cells were derived from patients with stage 4 tumors, suggesting that these cells are not representative of an ancestral state of the tumor but are derived from basal cancer cells.^[^ ^11^ ^]^ Except for these two patients, most cancer cells from most patients were of the basal subtype.

In the scRNA‐seq dataset, 133 953 cells that passed stringent quality control^[^ ^12^ ^]^ (Supporting Information Dataset 4) were clustered and annotated according to marker gene expression (Figure 1B; and Supporting Information Dataset 5) into epithelial cells, endothelial cells, fibroblasts, myeloid cells, CD3^+^ T lymphocytes, B lymphocytes, and natural killer (NK) cells (Figure 1C). These cells were derived from different donors (Figure 1D) and different tissue types (Figure S3A, Supporting Information).

Epithelial cells were the predominant cell types in cancer (Figure 1C). CNV‐free cells from tumor and normal urothelial tissues tended to cluster together, while malignant cells with significant CNVs segregated into different clusters (Figure 1E). This phenomenon is commonly observed in scRNA‐seq data of patients with cancer. Cancer cells were segregated into donor‐specific clusters (Figure 1F). Further annotation of these cancer cells based on marker gene expression revealed that the non‐proliferating cancer cells could be classified into basal and luminal classes (Figure 1G) based on the expression of *KRT7*, marker of basal cells, and *GATA3*, marker of luminal cells, respectively (Figure 1I). Based on the expression of cell cycle‐related genes (*MKI67* and *PCNA*) and the cell cycle scores (Figure 1H; Figure S3B, Supporting Information), the following two types of proliferating cancer cells were identified: a fast‐proliferating cancer cell subpopulations (FPCS) associated with each donor‐specific cluster (Figure 1G) and a unique cancer cell cluster predominantly comprising proliferating cells (Figure 1G). TM4SF1 positive cancer cell subpopulation (TPCS) was characterized by the upregulation of *TM4SF1*, *ITGA6*, and *KRT6A* (Figure 1I).

To analyze the contribution of cell type to the overall survival (OS) of patients, Scissor^[^ ^13^ ^]^ was used to analyze patient survival and RNA‐seq data from the BLCA Dataset of The Cancer Genome Atlas (TCGA).^[^ ^14^ ^]^ Among all cancer cells (basal, luminal, FPCS, and TPCS), TPCS exhibited the highest correlation with poor OS (Figure S3C, Supporting Information), suggesting that TPCS is strongly correlated with malignancy.

### 2.2 TPCS is a Shared Cancer Cell Type Among Patients with BLCA

**Figure 2:**
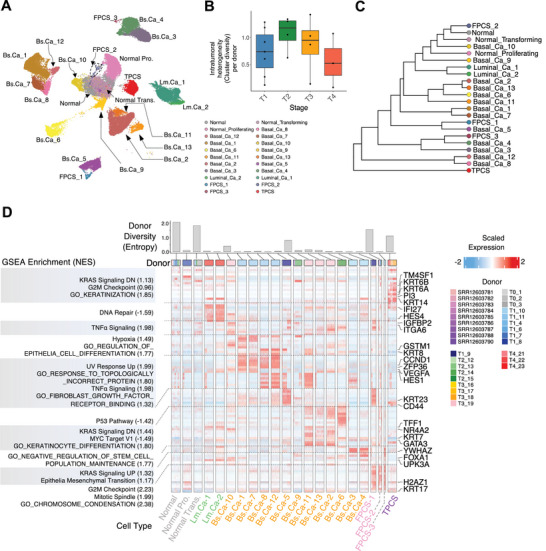
TPCS is a shared cancer cell type across patients. A) UMAP projection of scRNA epithelial cells. B) Donor‐wise epithelial cell type diversity across different clinical stages, showing apparent increase from T1 to T3 then decrease in T4. C) Phylogenetic tree of single cell clusters built by gene expression profile similarity. FPCS are always closely associated with a cancer or non‐cancer cluster, while TPCS occupies a unique branch. D) scRNA gene expression (heatmap), gene expression module by similarity clustering (dashed lines), GSEA enrichment of each gene expression module (left), and donor diversity (top column graph) of the scRNA epithelial cell clusters. Donors for each single cell are labelled on top color‐bar of the heatmap. Genes‐of‐interest are labelled on the right of heatmap. Donor/patient ID was designed by combing the tumor stage of a donor with a sequential identification number.

To examine the cancerous epithelial cells, the scRNA‐seq data were segregated and annotated to 22 single cell clusters based on their gene expression profile (**Figure** **2**A,D). These included 4 clusters dominated by normal cells (Normal, Normal Proliferating, Normal Transforming, and FPCS‐2), 15 clusters dominated by basal cancer cells (Basal_Ca_1‐13, FPCS‐1, and FPCS‐3), 2 clusters dominated by luminal cancer cells (Luminal_Ca_1‐2), and a single cluster dominated by TPCS (Figure 2A,D). Phylogenetic tree built based on the gene expression profile similarity revealed that most FPCSs are closely associated with a cancer or a non‐cancer cell cluster from the same donor and may represent a mitotic cell state of the associated cell cluster (Figure 2C).

Diversity of cancer cell clusters in the same patient increases during T1‐T2 stages and subsequently decreases in T3 and T4 stages, which suggests that ITH increases the initial outgrowth of the tumor clone but subsequently decreases due to selection or competition (Figure 2B). Gene Set Enrichment Analysis (GSEA) of the cluster‐specific gene expression profile revealed that cancer cells exhibited extensive gene expression heterogeneity even within a single patient. For example, cancer cells in patient T3‐19 segregated into four clusters (Basal_Ca_10, 11, 13, and 2) (Figure 2D). The most normal‐like cancer cell cluster Basal_Ca_10 exhibited upregulated TNFα pathway activity. In addition to the upregulated TNFα pathway activity, the Basal_Ca_11 cluster also exhibited upregulated keratinocyte differentiation pathway activity, which is shared among the resting clusters Basal_Ca_13 and Basal_Ca_2. Similarly, cancer cells in patient T1‐10 were segregated into four clusters (Basal_Ca_1, 7, 8, and 12) (Figure 2D), which exhibited differential pathway activity in hypoxia and epithelial cell differentiation.

Extensive ITH is a common hallmark in most patients with cancer. Thus, most cancer cell clusters are donor‐specific as evidenced by cluster‐wise donor diversity (Figure 2D). In contrast to the normal epithelial cells (Normal, Normal‐Trans, and FPCS‐2), most cancer cell clusters exhibited low donor diversity. Transcriptional ITH in early stage (T1‐T2) tumors is higher than that in late stage (T3‐T4) tumors (Figure 2B).

The TPCS cluster comprised only single cells from different donors, exhibiting the highest cluster‐wise donor diversity (Figure 2A,D). The intermixing of cells from different patients is not only a result of cell cycle‐related effects, but also TPCS uniquely expressed a set of non‐cell‐cycle‐related marker genes, such as *KRT6A*, *KRT6B*, *KRT14*, and *PI3* and exhibited unique upregulation of pathways related to keratinization and KRAS signaling (Figure 2D). Collectively, these results support the idea that TPCS is a shared cancer cell type among patients with BLCA and highlight the presence of transcriptomic ITH in BLCA.

### 2.3 TPCS is a Common Waypoint in BLCA Epigenomic Evolution

Transcriptional heterogeneity among BLCA tumor cells obfuscates the underlying shared molecular mechanisms driving their evolution (Figure 2). However, the stability of epigenetic modifications, such as DNA methylation (DNAm) and chromatin accessibility (ChrAcc) to track lineage development was higher than that of gene expression profiles. For example, DNAm patterns have been used to infer the evolutionary trajectory in clonal hematopoiesis.^[^ ^15^ ^]^ Probing DNAm at the single cell level is challenging. However, ChrAcc on differentially methylated regions (DMRs) is easily achievable. We hypothesized that differential DNAm could affect local ChrAcc, which enables the tracking of single cell development trajectory through ChrAcc on DMR. Thus, BLCA and normal bladder tissues were subjected to comprehensive genome‐wide DNAm sequencing and scATAC sequencing analyses.

To identify DMRs specific to BLCA, the data of MIBC and NMIBC, as well as those of BLCA and normal tissue samples, were comparatively analyzed (Supporting Information Dataset 6). Non‐hematopoietic lineage cells in the scATAC dataset were subsequently clustered based on their ChrAcc on CNV‐free DMRs. Following quality control, a total of 376,740 cells remained in the scATAC dataset, with 107,380 being epithelial cells. Fifteen clusters of single cells, each exhibiting distinct ChrAcc profiles on CNV‐free DMRs (Supporting Information Dataset 7), were identified and labeled “epigenotypes” (Figure S4A, Supporting Information Dataset 8). Through an analysis of ChrAcc on marker gene promoters, we determined that cells in epigenotype clusters 1–4 were stromal (mostly fibroblast) cells that showed elevated ChrAcc around *ZEB2* loci, while clusters 5–15 were epithelial cells with elevated ChrAcc in *GATA3* and *KRT6A* (Figure S4B, Supporting Information).

**Figure 3:**
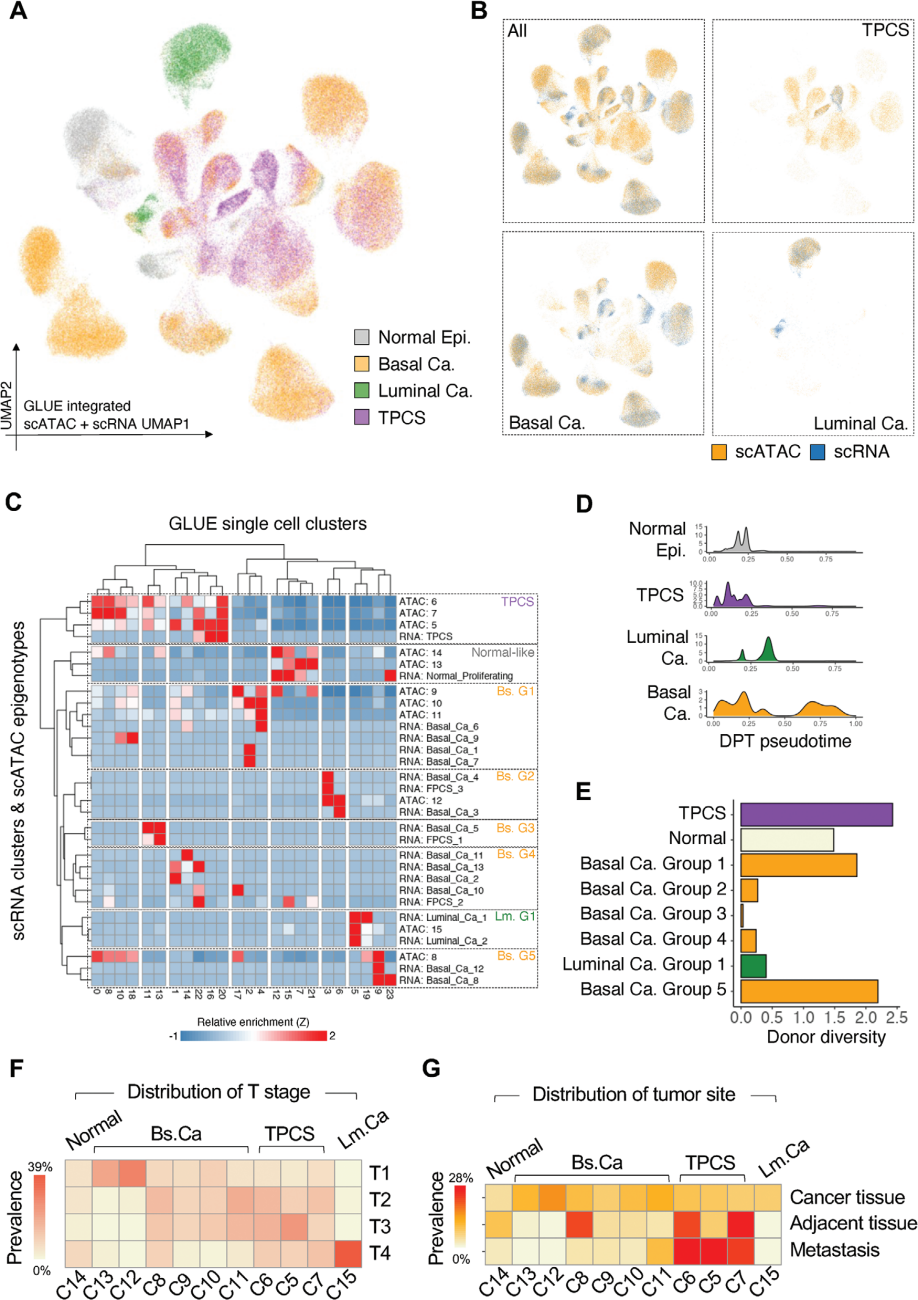
TPCS is a common waypoint in BLCA epigenomic evolution. A) UMAP from multimodal integration of scATAC and scRNA epithelial cells from BLCA samples. Cell types are denoted by color (normal: gray, basal cancer: orange, luminal cancer: green, TPCS: purple). B) Intercalation of scATAC and scRNA cells in the multimodal integration dataset. scATAC cells are orange and scRNA cells are blue. C) Heatmap of relative enrichment of each scATAC epigenotype or scRNA cell type (rows) in each cell cluster in the multimodal integration dataset (columns: GLUE clusters). Unsupervised hierarchical clustering results in 8 main cell groups: Normal, basal cancer G1‐G5, luminal cancer G1, and TPCS. Known scRNA and scATAC cells of similar type are clustered together. D) Pseudotime distribution of each major cell type in the multimodal integration dataset. E) Donor diversity of each cell group in the multimodal integration dataset. F) Heatmap of epigenotype cluster frequency across T stage. TPCS clusters (C5/C6/C7) are enriched in advanced T stage patients. G) Heatmap of epigenotype cluster frequency across tissue types. TPCS clusters (C5/C6/C7) are enriched in metastatic tissues and cancer adjacent tissues.

We then performed multimodal integration of scRNA and scATAC cells using GLUE^[^ ^16^ ^]^ (**Figure** **3**A). scATAC cells were fully intercalated in scRNA cells (Figure 3B), and cells from the similar donors were grouped together (Figure S5, Supporting Information). Using an independent multimodal integration method with ArchR,^[^ ^17^ ^]^ we matched scATAC cells to the most likely corresponding scRNA cell. Both orthogonal methods identified basal cells in the normal urothelium corresponding to Cluster 14, RNA‐determined TPCS cells corresponding to Clusters 5–7, basal‐like cancer cells corresponding to Clusters 8–13, and luminal‐like cancer cells corresponding to Cluster 15 (Figure 3C; Figure S4A, Supporting Information). Putative cancer cells showed increased chromatin accessibility on metastatic‐BLCA‐specific ATAC peaks (Figure S4C, Supporting Information).

Pseudotime trajectory analysis of scATAC data suggested that all cancer epithelial cells developed from normal‐like cluster14 cells (Figure S4A, Supporting Information), consistent with a scenario in which all MIBC cells share a common origin. In the multimodal integrated dataset, pseudotime analysis of the inferred developmental trajectory (Figure S6, Supporting Information) revealed that TPCS had the lowest pseudotime compared to luminal or basal cancer cells (Figure 3D), indicating that TPCS might be a common waypoint during BLCA evolution.

For the scATAC epigenotypes, we found high cluster‐wise donor diversity within the cancer‐associated epigenotypes (Figure S4D, Supporting Information), indicating that compared to scRNA clusters (Figure 2A,D), epigenotypes are less affected by ITH. TPCS epigenotypes show higher donor diversity compared to basal and luminal cancer cells (Figure S4D, Supporting Information). In the multimodal integrated dataset, we found that both scATAC and scRNA TPCS cells showed the highest donor diversity compared to other cancer cell types (Figure 3E), confirming that these cells are shared between patients.

A gradual shift in the epigenotype was observed across tumor stages. The C13 and C12 epigenotypes were relatively enriched in T1 tumors, while the C8, C9, C10, and C11 epigenotypes were enriched in T2 and T3 tumors. TPCS‐associated C5, C6, and C7 epigenotypes, as well as the luminal cancer C15 cluster, were enriched in T4 tumors (Figure 3F). A similar trend of shift from basal cancer cells toward TPCS was observed in all patients of the study cohort (Figure S7A, Supporting Information). Consistent with this result, TPCS was relatively enriched in extratumoral tissues, such as cancer‐adjacent tissue and metastatic sites (Figure 3G).

To analyze the diversity between TPCS epigenotypes, differentially accessible ATAC peaks between these clusters were computed (Figure S7B, Supporting Information). TPCS epigenotypes showed variability at differentially accessible regions that distinguished luminal and basal cancer types (Figure S7C, Supporting Information). This suggests that it might correspond to the multiple lineage tumorigenic progenitor cell type identified previously in murine models.

We noticed that in the multimodal integration dataset, scRNA clusters tended to segregate into different spatial positions, while scATAC epigenotypes intercalated between spatial clusters (Figure S8A, Supporting Information), suggesting that the epigenotype can bridge heterogeneous transcriptional states. If the evolution of BLCA transcriptional heterogeneity is driven by changes in epigenotypes, it is expected that transcriptional cell‐state transitions could only be bridged by epigenotypes. To test this hypothesis, we generated an adjacent matrix of scRNA single cell clusters and scATAC epigenotypes, considering their coexistence in similar clusters in the multimodal integration Dataset suggesting possible state transition. We then constructed a connectivity graph between these single cell states based on the adjacency matrix. On this connectivity graph, the connections between single cell clusters represent potential routes for state transition (Figure S8B, Supporting Information). Importantly, we found that the majority of these state transitions were facilitated by scATAC epigenotypes, with only a few direct RNA‐RNA transitions observed (Figure S8B, Supporting Information). scATAC epigenotypes showed many more transition connectivity's than scRNA clusters (Figure S8C, Supporting Information), with most transition connectivities attributed to TPCS (Figure S8D, Supporting Information). If we removed scATAC epigenotypes, the scRNA‐only graph would be fragmented into multiple isolated subgraphs and unconnected nodes, indicating that direct transformation between transcriptional states is unlikely. These results suggest that the evolution of BLCA is driven by changes in epigenotypes, particularly those corresponding to TPCS.

### 2.4 TPCS Emergence is Driven by EMT

**Figure 4:**
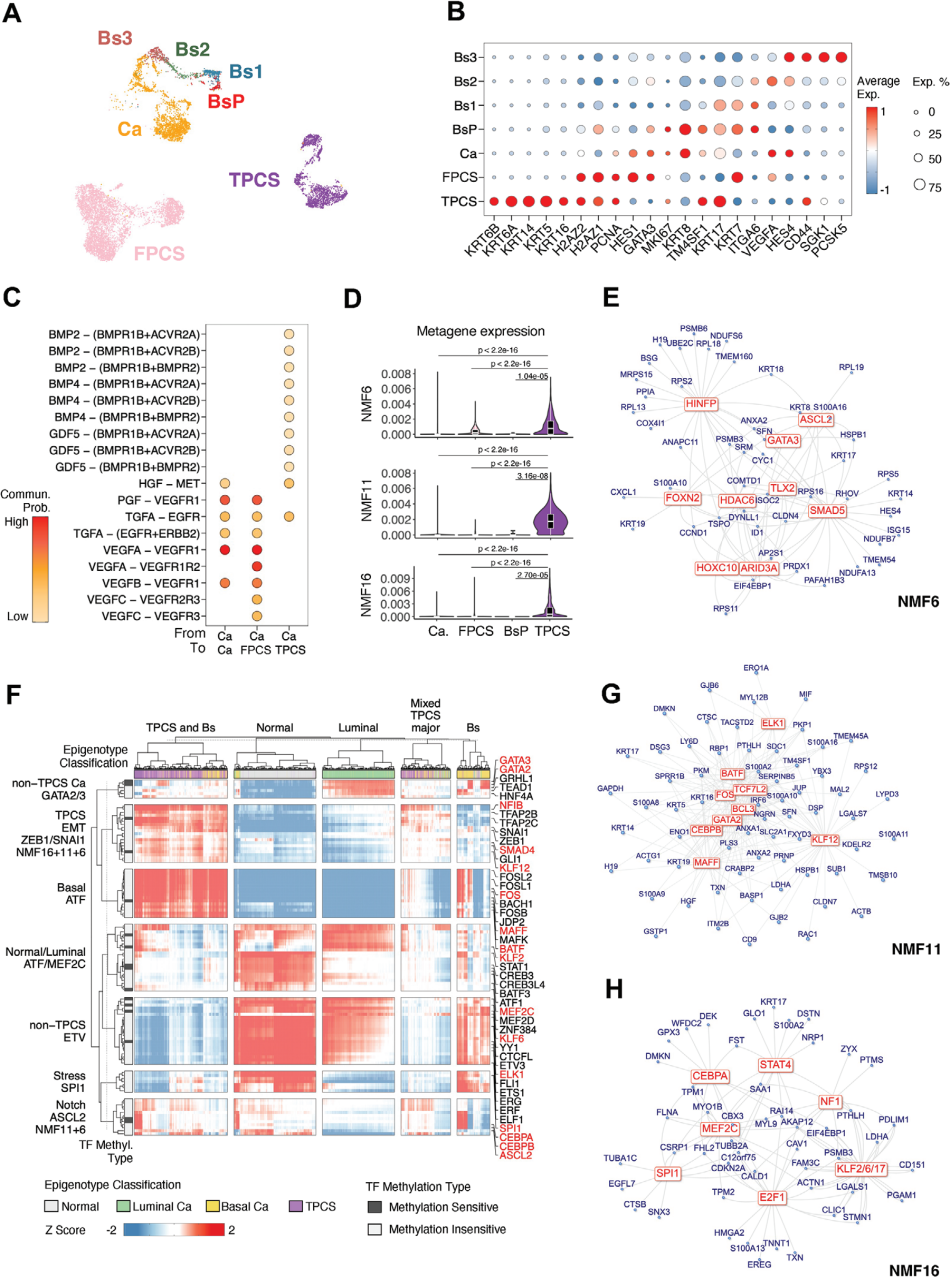
TPCS emergence is driven by BMP signaling induced EMT. A) scRNA cells projected to UMAP for this analysis, including normal urothelium basal cells (BsP, Bs1, Bs2, Bs3), basal cancer cells (Ca), FPCS, and TPCS. B) Differential expression of selected marker genes in scRNA cell types as in A). C) Cell–cell signaling probability between BLCA cells. Ca: Cancer. BMP signaling distinguished TPCS from cancer and FPCS. D) Non‐negative matrix factorization (NMF) component (“metagene”) expression in single cells. NMF component 6, 11 and 16 are selectively upregulated in TPCS. *P*‐value: wilcox test. E) Co‐regulated transcription networks extracted from NMF6 metagene set, with the putative common transcription factor that controls network‐wide gene expression (red) and target genes (blue). F) Genome‐wide chromatin accessibility inferred transcription factor activity in corresponding scATAC single cells. Cell class are color‐labelled on the top row of heatmap. Unsupervised clustering shows motif activity‐driven classification of these cells into 5 different groups: TPCS, Normal, Luminal, mixed, and Basal (Bs). Key transcription factors are noted on the right. Transcription factors in TPCS‐specific NMF6/11/16 are colored in red. Transcription factor clusters based on their activity profile across single cells are shown on the left. DNA methylation sensitivity of each transcription factor was shown in the left side bar. G) Co‐regulated transcription networks extracted from NMF11 metagene set. H) Co‐regulated transcription networks extracted from NMF16 metagene set. *P*‐value: t‐test.

To further understand the generation of TPCS, the scRNA expression data of normal urothelium basal cells (BsP, Bs1, Bs2, and Bs3, Figure S9, Supporting Information) and cancer cells (FPCS, TPCS, and basal cancer cells “Ca”) associated with the same class of sample that are strictly mappable to phenotypically similar scATAC clusters were re‐analyzed (**Figure** **4**A).

The scRNA expression profiles of these cells confirmed that BsP, Ca, FPCS, and TPCS are proliferative (*H2AZ1*/*H2AZ2/PCNA*/*MKI67* in Figure 4B). Compared with those in FPCS, Ca, and BsP, the expression levels of *TM4SF1*, *CD44*, and a set of keratins (*KRT6A*/*KRT6B*/*KRT5*/*KRT14*/*KRT16*) were upregulated, and the *HES1*/*HES4* expression levels were downregulated in TPCS (Figure 4B). This expression profile is distinct from that of other basal cancer cells but was similar to that of intermediate/umbrella cells in healthy urothelium (Figure S9C, Supporting Information). The overexpression of *TM4SF1* and keratin‐encoding genes in TPCS along with the downregulation of Notch downstream effectors and the potential loss of basal properties suggest a plasticity in the cell phenotype, which may allow TPCS to differentiate into multiple cell types and potentially contribute to the observed ITH in MIBC. The upregulation of *CD44* and the downregulation of *ITGA6* in TPCS also suggested a stem‐like phenotype, which may contribute to its ability to generate diverse cancer cell progeny. Cell‐cell signaling pattern between basal cancer cells (Ca), FPCS, and TPCS revealed that while Ca‐Ca and Ca‐FPC signaling mainly comprised PGF, TGFA, and VEGFA signaling, TPCS uniquely received BMP signals (BMP2/4 and GDF5) from Ca (Figure 4C).

Next, non‐negative matrix factorization (NMF)^[^ ^18^ ^]^ was performed to identify putative BMP‐responding TPCS‐specific gene expression profiles (Supporting Information Dataset 9). Three identified metagene modules (NMF6, NMF11, and NMF16) that were upregulated in TPCS cells relative to other cell types (Figure 4D,E,G,H). These metagene modules were found to be co‐regulated by transcription factors, such as SMAD, ASCL2, GATA2/GATA3, MEF2C, and BATF, because the promoter regions^[^ ^19^ ^]^ of genes within these modules were found to be enriched with transcription factor binding sites (TFBSs) of these co‐regulating transcription factors. For example, the binding site of SMAD5, a transcription factor that is directly downstream of BMP, is enriched in promoters of NMF6 genes (Figure 4E).

To validate the transcriptional regulation of these metagene modules, the activity of potential master transcription factors (TF) was evaluated by measuring the global transcription factor occupation footprint in scATAC data (Figure 4F). Cells were segregated into normal, luminal cancer, basal cancer (Bs), and TPCS. The activity of GATA‐family and MEF2C TF was upregulated in luminal cancer cells, while that of the ATF (BATF/MAFK/MAFF/BACH1) TF was upregulated in basal cancer cells. In contrast, TPCS cells were characterized by the downregulation of ETS family (ERG, ETS1, ELF1, and ETV3) TF activity and the upregulation of EMT‐related (NFIB, SNAI1, ZEB1, SMAD4) TF activity, which may be mediated via BMP signaling.

DNAm is known to impact transcription factor binding in vivo.^[^ ^20^ ^]^ Given that epigenome reprograming, particularly DNA hypomethylation, accompanied TPCS emergence (Figure S10, Supporting Information), we wondered whether DNAm could result in the observed differential transcription factor activity in TPCS. We stratified TFs into putative methylation‐sensitive or methylation‐insensitive classes.^[^ ^19a^ ^]^ No significant enrichment was found for methylation‐sensitive TFs in any groups of TFs segregated by their differential activity between BLCA single cells (Figure 4F), suggesting that DNA hypomethylation is unlikely to be upstream of differential TF activity. Hence, we consider TPCS generation to be primarily driven by EMT.

### 2.5 Malignant Transformation of TPCS from *Tm4sf1* Positive Intermediate Progenitor

**Figure 5:**
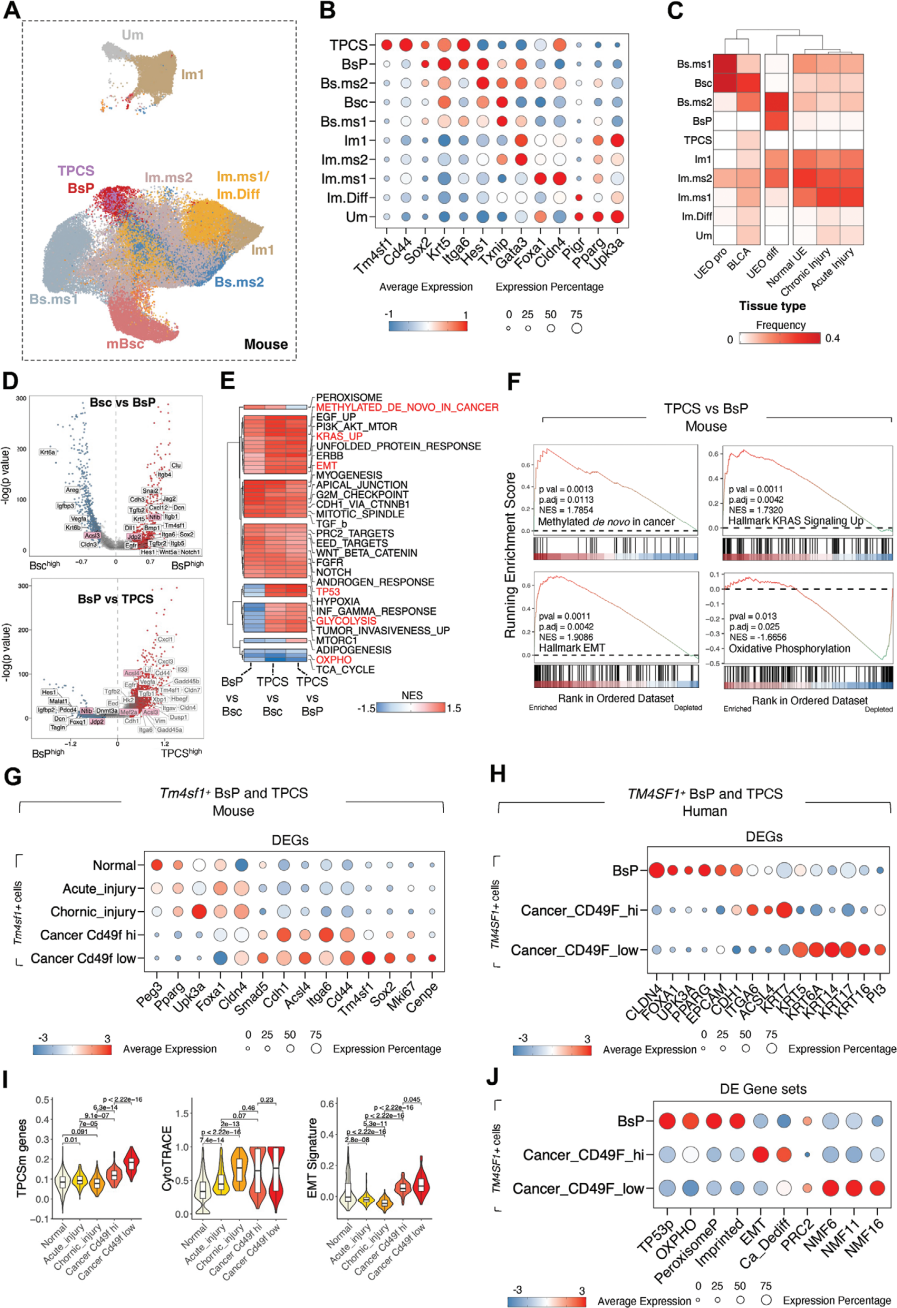
Malignant transformation of TPCS from Tm4sf1 positive intermediate progenitor. A) UMAP‐projection of mouse urothelium‐derived cells from mouse scRNA‐seq datasets including normal mouse urothelium (normal UE), acutely and chronically injured mouse urothelium (injured UE), *Cd49f* ^+^ mouse urothelium organoid in proliferating culture condition (UEO pro) or in differentiating culture condition (UEO diff), and OHBBN‐induced mouse muscle‐invasive bladder carcinoma (BLCA). Single cells were annotated with known cell markers according to human bladder urothelium counterparts. Bsc: bladder stem cell; Bs.ms1 and Bs.ms2: basal cells; BsP: *Tm4sf1* ^+^ cells in normal urothelium and organoids; TPCS: *Tm4sf1* ^+^ cells in OHBBN‐induced BLCA; Im1, Im.ms1, Im.ms2: intermediate cells; Im.Diff: differentiated intermediate cells; Um: umbrella cells. B) scRNA expression of selected marker genes within cell types in (A). TPCS is identified by *Tm4sf1*, *Sox2*, *Itga6*, *Cd44*, and *Hes1* expression but does not show lineage‐commitment markers expression, such as *Txnip*, *Foxa1*, *Cldn4*, *Pigr*, and *Upk3a*. C) Single cell prevalence of each cluster in different conditions. Bsc and Bs.ms1 enriched in proliferating UEO (UEO pro), while BsP and Bs.ms2 are enriched in differentiating UEO (UEO diff). Mouse OHBBN‐induced muscle‐invasive bladder cancer (BLCA) is enriched with both Bsc and TPCS. D) Upper panel: DEG between BsP and Bsc; lower panel: DEG between TPCS and BsP. Genes highlight in pink represent mouse homolog of human TPCS genes in NMF6/11/16. E) Gene set activity enrichment analysis of the DEG between Bsc, BsP, and TPCS in mouse. F) Gene expression contrast leading edge of selected gene sets between TPCS and BsP. G) Gene expression profiles of *Tm4sf1* ^+^ cells from different tissue types in mouse. H) Gene expression profiles of human *TM4SF1* ^+^ cells from normal bladder (BsP) and BLCA tissues. I) TPCS and EMT metagene score and cell stemness (CytoTRACE) of mouse *Tm4sf1* ^+^ cells across different tissue types. J) Gene set expression scores of human BsP and TPCS (Cd49f^high^ or Cd49f^low^). Abbreviations: TP53p: TP53 pathway; OXPHO: oxidative phosphorylation pathway; PeroxisomeP: peroxisome pathway; Imprinted: known imprinted genes; Ca_Dediff: known de‐differentiation gene set for cancer. *P*‐value: t‐ test with BH adjustion.

Two alternative scenarios could explain TPCS enrichment in advanced stage BLCA. In the first case, TPCS can gradually emerge only in the late stage. Meanwhile, in the second case, TPCS can arise in the early stage but is selected in the late stage. Although our bioinformatic analysis suggested the first scenario, we did not obtain direct evidence from early‐stage human BLCA samples. Obtaining early‐stage samples of human BLCA is challenging, hence, the published scRNA sequencing data from the bladder cancer‐related mouse model, including data on non‐cancerous bladder urothelium tissue in normal or injured conditions,^[^ ^21^ ^]^ bladder organoids (organoid),^[^ ^22^ ^]^ and N‐butyl‐N‐4‐hydroxybutyl nitrosamine (OHBBN)‐induced BLCA,^[^ ^11^ ^]^ were re‐analyzed. scRNA‐seq data integration (**Figure** **5**A) and annotation based on gene expression (Figure 5B) revealed major epithelial cell types, including the basal stem cells (Bsc), basal cells (Bs.ms1 and Bs.ms2), intermediate cells (Im1, Im.ms1, Im.ms2, and Im.Diff), and umbrella cells (Um) (Figure 5A,B).

Mouse Bsc, the stem cell population in the normal and proliferative organoid datasets, was marked by high *Krt5* expression, and low *Gata3*/*Foxa1*/*Cldn4* expression (Figure 5B,C). Additionally, a *Tm4sf1* ^+^ cell population was identified in the mouse bladder organoid scRNA‐seq dataset. This population exhibited transcriptional similarity to human BsP in normal urothelium (Figure S9B, Supporting Information), as indicated by elevated expression of *Tm4sf1*, *Krt5* and *Cd49f* (*Itga6*, homolog to human *ITGA6*), and reduced *Cd44* expression compared to Bsc. We termed the mouse *Tm4sf1* ^+^ cells in normal tissues (organoid) as mouse BsP. Pseudotime analysis suggested that mouse BsP are directly derived from Bsc and predate the generation of basal, intermediate, and umbrella cells in mouse urothelium organoids (Figure S11A, Supporting Information). A *Tm4sf1* ^+^ cell population was also identified in mouse BLCA, which not only expressed *Tm4sf1* but also showed further elevated expression levels of *Tm4sf1*, *Cldn4*, *Cd44*, *Cd49f*, and *Sox2* compared to BsP (Figure 5B). This is partially similar to TPCS in human cancer which overexpressed *CD44* and *TM4SF1*. We term the *Tm4sf1* ^+^ cells in cancerous tissues as mouse TPCS.

In the organoid model, the removal of essential compounds, such as Wnt agonist, TGFβ inhibitor, Noggin, and EGF from the maintenance medium forces the organoid to switch from a “proliferating” state (UEO^pro^) into a “differentiating” state (UEO^diff^).^[^ ^22^ ^]^ Bsc and Bs.ms1 were significantly enriched in UEO^pro^ (Figure 5B,C). In contrast, these cells are depleted in differentiating UEO^diff^ and replaced by BsP and Bs.ms2 (Figure 5B,C). These results indicate that *Tm4sf1* marks a naturally existing intermediate progenitor population in normal uroepithelium.

Differentially expressed genes (DEGs) between normal urothelium *Tm4sf1* ^+^ cells (BsP), malignant *Tm4sf1* ^+^ cells from cancer (TPCS), and normal urothelium Bsc (Figure 5D) were analyzed. DEGs corresponding to the human BLCA TPCS‐specific transcription module NMF6/11/16 were highly likely to be enriched in TPCS or BsP (pink genes in Figure 5D). Compared with Bsc, BsP exhibited an increased abundance of EMT‐related genes, such as *Snai2* and *Dcn* (Figure 5D upper panel). Conversely, TPCS exhibited upregulation of *Il33*, *Gadd45b*, and *Eed* (a PRC2 complex member) compared with BsP (Figure 5D lower panel). Stepwise GSEA was performed to characterize the gene expression profiles of Bsc, BsP, and TPCS (Figure 5E). Compared with Bsc, BsP exhibited upregulation of EGF/ERBB pathway‐related and PRC2 target genes. This suggests that these cells are intermediate progenitors in the transformation process. The activities of both oxidative phosphorylation and glycolysis were downregulated in BsP, indicating that it is metabolically inert. Compared with BsP, TPCS was associated with the upregulation of genes involved in glycolysis and hypoxia, and mTOR signaling (Figure 5E). Gene set activities that were significantly upregulated in TPCS compared to BsP included “Methylated de novo in cancer”, “KRAS signaling UP”, and “EMT”, while “Oxidative phosphorylation” was downregulated in TPCS (Figure 5F), suggesting that the malignant transformation from BsP toward TPCS involves metabolic and epigenomic reprograming downstream of enhanced MAPK signaling and EMT.

We performed detailed analysis of gene expression profiles in mouse *Tm4sf1* ^+^ cells, including BsP and TPCS, across normal tissue, injured tissue, and cancer (Figure 5G). *Itga6* expression levels can drift in mouse BLCA cells, and are inversely correlated with their tumor‐generation capacity in mouse BLCA allograft/xenograft models.^[^ ^11^ ^]^ The *Tm4sf1* ^+^ TPCS in cancer were naturally segregated into two populations with low and high *Itga6*/*Cd49f* expression by unsupervised clustering, suggesting phenotypic heterogeneity in this population (Figure S11B,D, Supporting Information). Similar to human TPCS, the expression level of the BMP downstream TF *Smad5* is increased in cancer TPCS compared to non‐malignant BsP (Figure 5G). Comparing with CD49f^high^ TPCS, the higher expression of *Smad5*, *Sox2*, *Cldn4*, and lowered expression of *Foxa1* in CD49f^low^ TPCS (Figure 5G) suggest that it has undergone complete transformation. Gradual transformation from normal urothelium BsP toward Cd49f^low^ TPCS is possibly bridged by the injury‐induced state, as these cells showed intermediate levels of *Cldn4* and the imprinted gene *Peg3* expression. Indeed, single cell stemness (CytoTRACE) analysis of the *Tm4sf1* ^+^ cells showed that urothelium injury induced cell dedifferentiation (Figure 5I), which may prime EMT in cancerous *Tm4sf1* ^+^ cells (Figure 5I).

Having determined that BLCA TPCS is a direct derivative of BsP in mice, we further compared the gene expression profiles of human *Tm4sf1* ^+^ cells, including BsP (Figure S9, Supporting Information) and TPCS. Like the mouse cancerous *Tm4sf1* ^+^ cells, human TPCS could further segregate into CD49F^high^ and CD49F^low^ cell populations (Figure 5H; Figure S11C,E, Supporting Information). Compared to BsP and CD49F^high^ TPCS, the CD49F^low^ TPCS specifically expressed a series of keratins (*KRT5*, *KRT6A*, *KRT14*, *KRT16*, *KRT17*) and *PI3* (Figure 5h). Gene set differential expression analysis showed that genes in the TP53, oxidative phosphorylation and peroxisome metabolism pathways were downregulated in TPCS compared to BsP (Figure 5J). Cancer‐associated dedifferentiation gene signature was strongly upregulated in CD49F^high^ TPCS,^[^ ^23^ ^]^ whereas PRC2‐controlled gene expression^[^ ^24^ ^]^ was lowest in CD49F^high^ TPCS (Figure 5J), confirming that it was transformed toward an undifferentiated state. On the other hand, strongly upregulated TPCS‐associated NMF metagene module activity (*NMF6*, *NMF11*, *NMF16*) in CD49F^low^ TPCS indicated that it was fully transformed (Figure 5J).

Taken together, these findings indicate that *Tm4sf1*/TM4SF1 marks an intermediate progenitor cell state in normal urothelium or organoids. The cancer TPCS is an early cancer cell state during tumorigenesis, that is transformed from naturally existing *Tm4sf1^+^* BsP cells by dedifferentiation.

### 2.6 Hyperplastic TPCS Generates ITH after EMT

**Figure 6:**
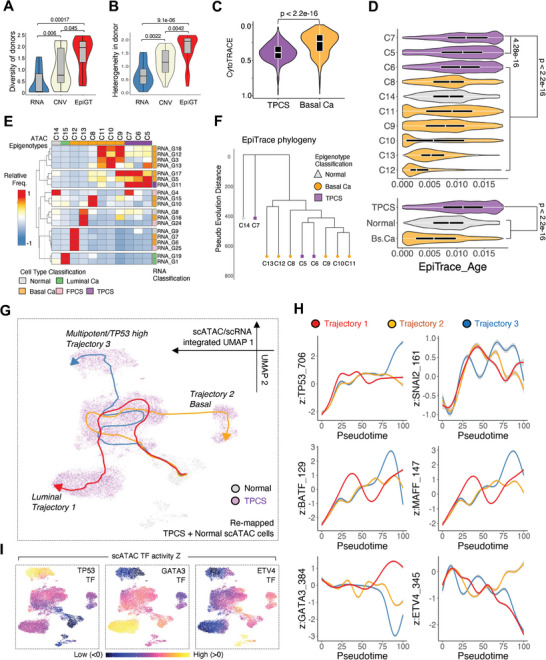
ITH generation from hyperplastic TPCS post‐EMT. A) Donor diversity of single cell clusters, classified by different methods. EpiGT: epigenotype; CNV: scATAC‐inferred copy number variation; RNA: transcription profile. Compared to CNV and RNA, epigenotypes are more widely shared among the donors. B) Heterogeneity of single cell clusters within each donor. Highest intratumoral heterogeneity is found for epigenotypes compared to RNA or CNV. C) CytoTRACE‐inferred differentiation state (0: differentiated, 1: stem‐cell‐like) of TPCS and basal cancer scRNA cells. D) EpiTrace‐inferred mitosis number of scATAC cells grouped by epigenotypes (top) or general cell class (bottom). E) Frequency of single scATAC cells of epigenotype (columns) matched to scRNA transcriptional phenotype classes (rows). Cell type classification for both epigenotype and transcriptional phenotype are colored in side bars (Normal, grey; Luminal cancer, green; Basal cancer, orange; FPCS, pink; TPCS, purple). F) EpiTrace constructed developmental phylogeny of epigenotypes. G) Developmental trajectory from normal epithelial cell toward TPCS in multimodal dataset. Grey, Normal cells; purple, TPCS; red, trajectory 1/luminal; orange, trajectory 2/basal; blue, trajectory 3/multipotent‐TP53 high. H) Transcription factor activity during normal‐to‐TPCS transformation along the inferred developmental trajectories (red: trajectory 1/luminal; orange: trajectory 2/basal; blue: trajectory 3/multipotent‐TP53 high). I) Visualization of transcription factor activity of single scATAC cells on UMAP. TP53 marks the terminal of multipotent trajectory, GATA3 marks the terminal of luminal trajectory, and ETV4 labels the basal trajectory. *P*‐value: Wilcoxon test.

In our scATAC dataset, epigenotypes are the most likely to be shared between patients/tumors, followed by CNV profiles and RNA transcription. This indicates that epigenotype is the shared core phenotype under extensive transcriptional heterogeneity (**Figure** **6**A). However, heterogeneity between epigenotypes within a donor is significantly higher than CNV profiles and RNA transcription (Figure 6B), indicating that the epigenotype shift drives BLCA evolution within a single BLCA population.

Most basal and luminal cancer epigenotypes matched only to the corresponding RNA cell type (Figure 6E). TPCS‐corresponding epigenotypes C5, C6, and C7 matched to not only the TPCS RNA cell type but also to several other cell types, mostly basal cancer cells (Figure 6E).

The extensive transcriptional ITH (Figure 2) in early stage BLCA and the gradual convergence toward the TPCS epigenotype in late stage (Figure 2 and Figure 3) suggest that TPCS might be linked to ITH generation. To examine whether TPCS is the core ITH generator cell population, orthogonal bioinformatic approaches were used to infer cell developmental lineage according to clock‐like ChrAcc^[^ ^25^ ^]^ and infer cell stemness by examining RNA expression diversity.^[^ ^26^ ^]^ The number of mitoses that a cell has undergone can be computationally inferred using ChrAcc on clock‐like differentially methylated loci (ClockDML). Furthermore, phylogenetic trees constructed using this method can be used to track cell lineage during development and oncogenesis.^[^ ^25^ ^]^ In combination with stemness inference, this method enabled lineage tracing (instead of trajectory inference) from single cell sequencing data with sufficient temporal resolution.

The scRNA profiles revealed that the stemness of TPCS was higher than that of basal cancer cells (Figure 6C). Consistent with this result, scATAC data revealed that the mitotic age in TPCS cells was higher than that in other basal cancer cells (Figure 6D), indicating that TPCS undergoes more mitosis than basal cancer cells. The phylogenetic tree revealed that cells of TPCS epigenotypes, especially C7, are placed next to normal urothelium epithelial cells (C14), suggesting that C7 cells are the first derived cells in the phylogeny (Figure 6F). The gradual decrease in the diversity of the matched RNA phenotype by TPCS epigenotypes C7 to C6 and C5 (Figure 6D) is consistent with the placement of C5 and C6 in distal branches of the phylogenetic tree.

We then analyzed the developmental trajectory leading to and from TPCS by detailed joint scATAC/scRNA integrated analysis. TPCS and normal epithelial cells in the scRNA and scATAC datasets, defined by GLUE clustering, were integrated (Figure S12A–C, Supporting Information). RNA expression profiles were inferred for each scATAC cell by multimodal integration. Pseudotime trajectory analysis showed that normal cells first developed into a “core TPCS” cell type, and then trifurcated into three different clusters (Figure 6G). Significant variations in TF activity in TP53, SNAI2, BATF, MAFF, GATA3, and ETV4 were found during the transformation (Figure 6H; Figure S12D–F, Supporting Information). We noticed that EMT‐associated, TPCS‐specific SNAI (Figure 4F) activity was commonly upregulated in all trajectories in the early phase of transformation (Figure S12D–F, Supporting Information), indicating that EMT is an initiator mechanism driving TPCS transformation. The basal‐cancer‐specific BATF and MAFF (Figure 4F) activities were monotonically upregulated during transformation (Figure 6H, middle panel).

The three developmental trajectories differ after EMT (Figure 6G): TP53 activity is upregulated in trajectory 3 (Figure 6I), luminal‐specific GATA3 (Figure 4F) activity is uniquely upregulated in trajectory 1 and downregulated in others (Figure 6I), and basal‐specific ETV (Figure 4F) activity is uniquely upregulated in trajectory 2 (Figure 6I). Detailed analysis of transcription factor expression and their activity along these trajectories showed fate‐priming transcriptional events for trajectory selection: RUNX1 and ELF2 activity during the intermediate development stage leads to multipotent trajectory 3, HES4 activity during the early‐to‐intermediate development stage leads to luminal‐priming trajectory 1, and FOXJ2 activity during the intermediate development stage leads to basal‐priming trajectory 2 (Figure S12, Supporting Information). These results reveal the molecular mechanisms determining post‐EMT TPCS development into adopt luminal (trajectory 1), basal (trajectory 2), or multipotent (trajectory 3) fates.

Cells from luminal‐fate‐priming trajectory 1 cells are mostly clinical stage T1, while T2 cells are enriched in basal‐fate‐priming trajectory 2, and T3/4 cells are enriched in multipotent trajectory 3, suggesting that the fate choice of TPCS determines tumor growth potential (Figure S12B, Supporting Information). Together, these results indicate that TPCS is the early‐emerging tumor initiator population that generates ITH in human BLCA.

### 2.7 Tumor‐Reactive T Cells in BLCA Implicate Continuous Selection on Cancer Cells

Mechanistically, ITH may facilitate cancer cell immune evasion by shifting neoantigen expression or directly upregulating immune suppression molecules. As TPCS arises early during oncogenesis and is enriched in late‐stage tumors, we hypothesized that TPCS is selected by immune surveillance. We comprehensively analyzed immune cells, including CD8^+^ T lymphocytes (Figure S13), CD4^+^ T lymphocytes (Figure S14, Supporting Information), NK cells (Figure S15, Supporting Information), B cells (Figure S16, Supporting Information), and myeloid cells (Figure S17, Supporting Information) in the scRNA dataset. Myeloid cells (cDC, M1, M2), NK cells, B IGHM cells and T cells (including CD4 T and CD8 T cells) were associated with BLCA patient survival in Scissor analysis (Figure S3C, Supporting Information). Among these putative candidate immune control cells, only M1/2 macrophages and T cells are enriched in cancer tissue (Figures S13H, S14I and S17G, Supporting Information). Adaptive immunity against tumors is primarily mediated by T lymphocytes. Thus, the tumor‐reactive population in intratumoral T lymphocytes in BLCA was characterized.

Previous reports have suggested that although some tumor‐reactive T cells may persist in the tumor, a significant proportion of tumor‐infiltrating lymphocytes (TILs) are non‐reactive, bystander T cells.^[^ ^27^ ^]^ To examine the tumor‐reactivity of intratumoral T cells in BLCA, the clonal expansion status of T lymphocytes was examined by analyzing the prevalence of TCR clone sequences across tissues. Additionally, the expression of tumor‐reactiveness marker genes on these cells was examined. Local clonal expansion of a T cell clone was defined by the observation of multiple single cells with similar TCR sequences within the tumor, whereas global clonal expansion was defined by the observation of multiple cells with similar TCR sequences in multiple tissues.

For the CD8^+^ T cell population, globally‐expanded cells including the immature, non‐ PDCD1 (PD‐1)‐expressing TEM clusters, PBMC‐Tem XCL, (KLRG1^+^, PRF1^+^, and XCL^+^ populations), as well as PD‐1 expressing actT_eff_ (GZMK^+^), do not express the tumor‐reactiveness markers *ENTPD1* (*CD39*) and *ITGAE* (*CD103*), and hence are considered non‐tumor‐reactive (Figure S13B, Supporting Information). In the *ENTPD1*/*ITGAE* double positive (tumor‐reactive, tr) population, local TCR clonal expansion and PD‐1 expression were used to classify the following four clusters of effector T cells (T_eff_): the TOP2A^+^ proliferating prT_eff_, IFNG^high^ trT_eff_, IFNG^low^ trT_eff_, and IFNG^−^/CXCR6^+^ trT_eff_ (trT_eff_‐CXCR6) (Figure S13B, Supporting Information). Compared with other tissues analyzed, these trT_eff_ were more prevalent in cancer tissues (Figure S13H, Supporting Information). Among these tumor‐reactive CD8^+^ cytotoxic effector T cells, trT_eff_‐CXCR6 is the only *PDCD1^+^/ENTPD1^+^/ITGAE^+^* population that contains globally‐expanded clones (Figure S13C, Supporting Information) and might support strong antitumor activity in both primary and metastatic tumor sites.^[^ ^28^ ^]^ In concordance with reports in other cancer types,^[^ ^28^, ^29^ ^]^ trT_eff_‐CXCR6 gene expression signature is associated with favorable clinical outcomes (Figure S13J, Supporting Information).

For CD4^+^ T cells, clonal expansion was highly prevalent in the mature T cell population (bsTh2/17, trTh1, cT_reg_, eT_reg_, and trT_reg_) (Figure S14A, B and D, Supporting Information). Among these CD4^+^ T cells, globally‐expanded populations, including bsTh2, bsTh17, and eT_reg_, do not express *ITGAE* and *ENTPD1* (Figure S14A–C, Supporting Information). In contrast, locally‐expanded trTh1 and trT_reg_ cells overexpress *ITGAE*/*ENTPD1* in addition to *RGS2*/*IRF1*/*TNFRSF4* (Figure S14B, Supporting Information).

**Figure 7:**
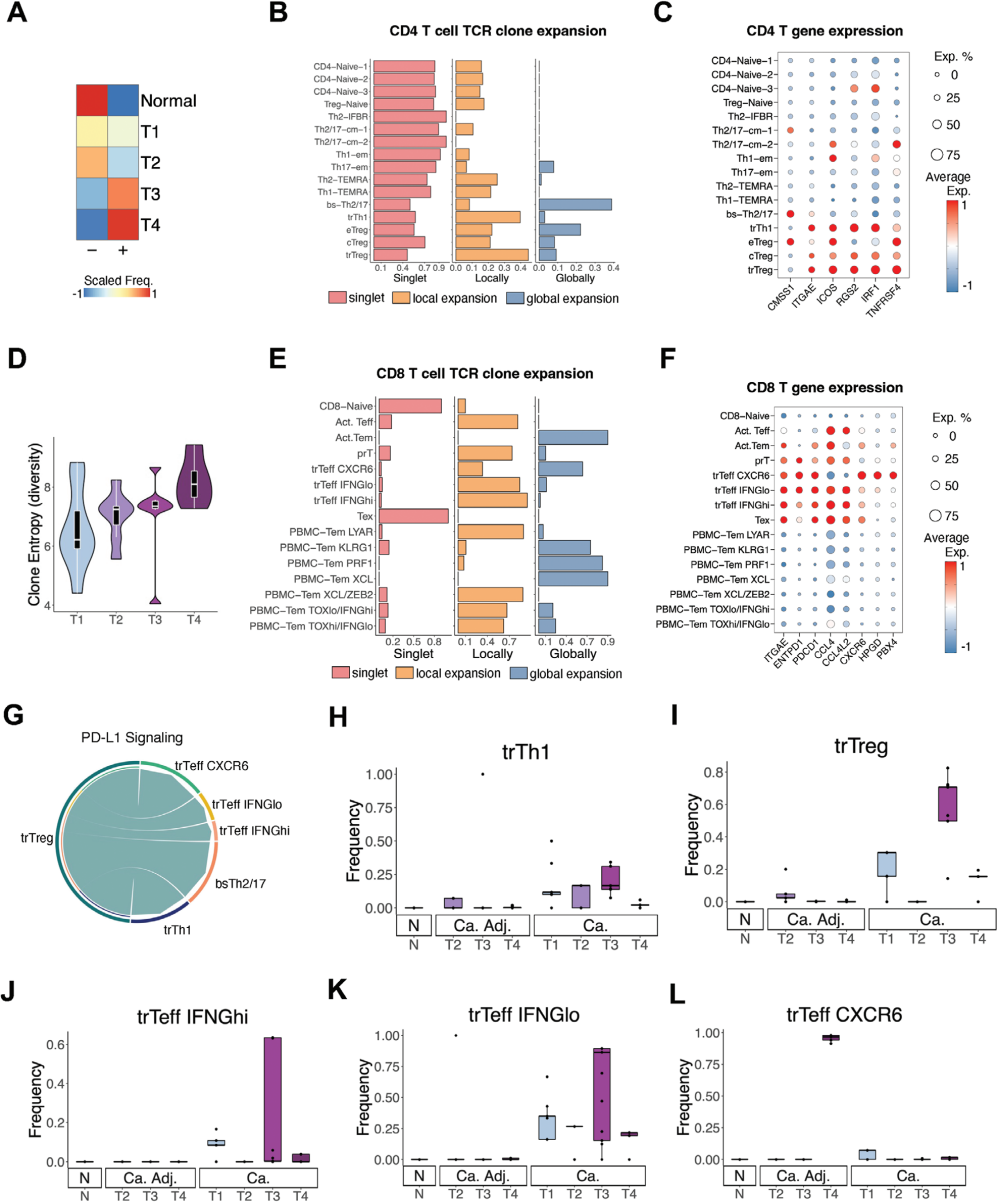
Tumor‐specific adaptive immunity in BLCA. A) Relative frequency of donors with (+)/without (‐) locally expanded TCR clone in each clinical stage. B) Frequency of CD4 T cells with singlet (non‐expanded) TCR (red), locally‐expanded TCR (orange), or globally‐expanded TCR (blue). C) Single cell expression of tumor‐reactiveness‐related genes among the CD4 T cell clusters. D) TCR clone diversity (entropy) in donors of different clinical stage. E) Frequency of CD8 T cells with singlet (non‐expanded) TCR (red), locally‐expanded TCR (orange), or globally‐expanded TCR (blue). F) Single cell expression of tumor‐reactiveness‐related genes among the CD8 T cell clusters. G) Cell–cell signaling analysis between immune cells showed that PD‐L1 signaling is mainly from trT_reg_ cells toward other tumor‐reactive T cells including trT_eff_, bsTh2/17, and trTh1. H–L) Relative cell frequency of trTh1 H), trT_reg_ I), trT_eff_‐IFNG^high^ J), trT_eff_‐IFNG^low^ K), and trT_eff_‐CXCR6 L) in donors of different clinical stages.

We analyzed relative frequency of donors with locally expanded TCR clone and TCR clone diversity (entropy) in each clinical stage of BLCA. The prevalence of T cells with(+)/without(‐) clonal expansion 675 across BLCA T stages. 100% advanced T stage BLCA patients have clonally expanded T cells in tumor tissue (**Figure** **7**A). TCR clonotype diversity (Shannon entropy) across clinical stages shows a general trend of increase in clonotype diversity in the advanced clinical stage (Figure 7D). Based on previous studies^[^ ^27^, ^30^ ^]^ and the observation of local TCR clone expansion (Figure 7B,E), the expression of *ITGAE*/*ENTPD1* was considered to serve as a general marker for tumor‐reactiveness also for these CD4^+^/CD8^+^ T cells (Figure 7C,F). Cell‐cell signaling analysis indicated that trT_reg_ contributed most of the PD‐L1 ligand to other T cells in the tumor environment (Figure 7G). Notably, there was an increase in the prevalence of trT_reg_ and trTh1 over clinical stage progression (Figure S14J, Supporting Information). We found that T lymphocyte clonal expansion was absent in healthy donors, but present in T1‐T4 donors (Figure 7H–L). Both the prevalence of clonally‐expanded T lymphocytes (Figure 7H–L) and the diversity of TCR clones (Figure 7D) were positively correlated with T‐stage, suggesting that real tumor‐reactive T lymphocyte responses persist in BLCA, as evidenced by robust T cell clonal expansion. These results indicate that the BLCA tumor microenvironment is not completely immunosuppressive and continues to attract and train new naïve T cells to become tumor‐reactive controllers. Hence, BLCA might evade immunosurveillance via alternative, cancer‐cell‐autonomous mechanisms.

### 2.8 Contribution of TPCS‐Generated ITH to Immunosurveillance Evasion

**Figure 8:**
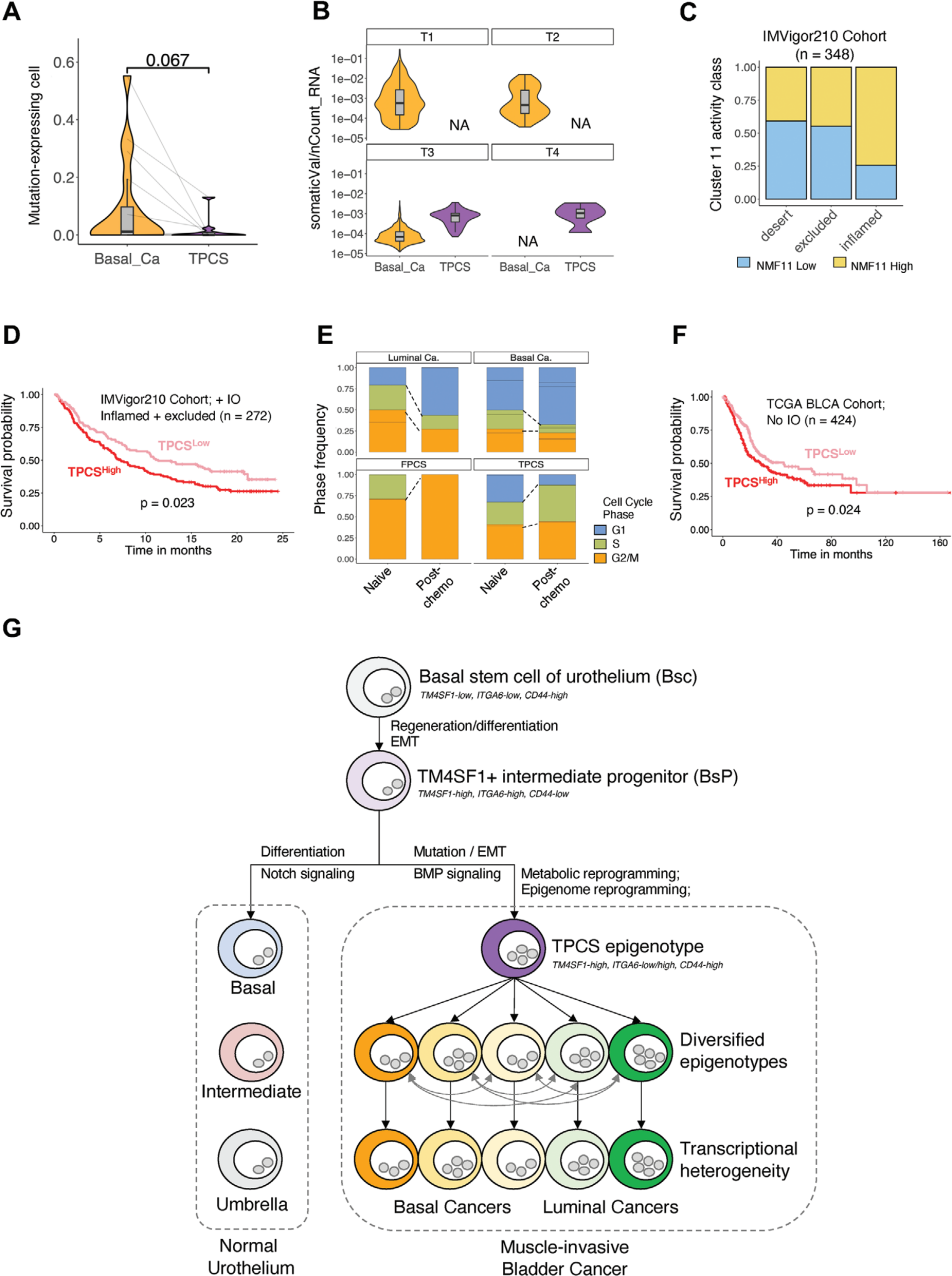
TPCS‐generated ITH underlies therapeutic resistance and mechanistic diagram of the study. A) Frequency of single basal cancer and TPCS cells expressing mutation‐carrying transcripts in each donor. B) Single cell mutation‐carrying transcript expression level of the mutation‐expressing cells for TPCS and basal cancer across clinical stages. N/A: not available. C) TPCS‐specific NMF11‐metagene expression class in BLCA of different immune categories: desert, excluded, or inflamed. D) Survival probability of patients with high and low TPCS‐specific NMF11 metagene activity in IMVigor210 cohort (immunocheckpoint inhibitor‐treated, IO). E) Cell cycle phase of luminal cancer, basal cancer, FPCS, and TPCS from naïve or post‐chemotherapy patients. F) Survival probability of patients with high and low TPCS‐specific NMF11 metagene activity in TCGA BLCA cohort where most patients are treated by chemotherapy. G) Main findings of the study. Normal urothelium develops from basal stem cell (Bsc) through intermediate basal progenitor (BsP) cells. In normal development, BsP cells derives into the basal, intermediate and umbrella cells of urothelium. During tumorigenesis, BsP cells undergone EMT to form a TM4SF1+ cancer cell subpopulation (TPCS). TPCS harbors a unique epigenome, which is hyperplastic and can develop into transcriptionally heterogenic bladder cancer cells. *P*‐value: t‐test (grouped) or Cox method (survival).

Next, MHC‐related molecule expression and somatic mutation‐carrying (“neoantigen”) transcript expression in cancer cell scRNA‐seq data were analyzed. The number of TPCS cells expressing somatic mutation‐carrying transcripts was lower than that of basal cancer cells (**Figure** **8**A). In basal cancer cells, the mutation‐carrying transcript expression level was inversely correlated with the clinical stage, suggesting that these cells expressing immunogenic peptides are targeted by immune selection. In contrast, TPCS cells stably expressed neoantigen transcripts across clinical stages (Figure 8B). These results suggest that TPCS switch between neoantigen‐high and neoantigen‐free states. This switch generates ITH of immunogenicity, which may aid cancer to evade immunosurveillance at a population level.

To validate this result, the correlation between TPCS and immunocheckpoint therapy response was examined. If TPCS cells actively evade immune selection, they may serve as a prognostic biomarker for immunocheckpoint inhibitor resistance. The IMvigor210 clinical trial^[^ ^31^ ^]^ included 119 patients with locally advanced or metastatic BLCA patients who had undergone immunocheckpoint inhibitor (ICI) therapy. TPCS prevalence in BLCA tumor tissues sequenced using the Imvigor210 RNA sequencing Dataset^[^ ^32^ ^]^ was quantified by measuring the gene set activity of the TPCS marker metagene NMF11. In this dataset, compared with immune‐desert tumors, inflamed tumors were more likely to contain increased numbers of TPCS (Figure 8C). In the group of patients who received ICI treatment, patients with high TPCS marker gene expression exhibited significantly poorer prognosis (Figure 8D), suggesting that TPCS contributed to ICI resistance.

### 2.9 Contribution of TPCS‐Generated ITH to Chemotherapy Resistance

In addition to facilitating evasion from immune selection, ITH may benefit cancer cell survival by facilitating drug resistance through the generation of a pool of diverse cancer cells that differentially respond to therapy. A subgroup of patients in the study cohort received adjuvant intravesical gemcitabine instillation. After chemotherapy treatment, most basal‐like and luminal‐like cancer cells tended to be arrested in G1 and did not enter S or G2/M phases (Figure 8E). Additionally, FPCs tended to be arrested in G2/M. However, the prevalence of both S and G2/M‐phase TPCS cells increased, suggesting that chemotherapy‐mediated TPCS cell cycle arrest was minimal (Figure 8E).

To validate the association of TPCS with BLCA chemoresistance, TPCS prevalence was quantified in the TCGA BLCA cohort. The cohort was classified into high TPCS and low TPCS subgroups. Patient survival was analyzed according to subgroups. In this cohort, patients were mainly treated with chemotherapy. High TPCS prevalence was significantly correlated with poor prognosis (Figure 8F), suggesting that TPCS contributed to chemotherapy resistance.

## 3 Discussion

Cell‐of‐origin of human BLCA: The mechanisms involved in the evolution of BLCA, a complex and heterogeneous disease, from normal urothelium to generate heterogeneity have not been elucidated.^[^ ^8a^ ^]^ Clinical observations have demonstrated that cancer recurrence is highly likely in patients with superficial papillary BLCA. Meanwhile, most patients with high‐grade invasive BLCA do not have a history of superficial papillary BLCA.^[^ ^33^ ^]^ Therefore, two distinct tracks, termed papillary and nonpapillary/high‐grade tracks, are postulated for BLCA progression.^[^ ^8^, ^34^ ^]^ Muscle‐invasive cancer cells originate from the basal cells in the urothelium of an animal model of MIBC induced by OHBBN.^[^ ^8b^ ^]^ One study speculated that the progenitors in NMIBC tumors originate from non‐basal cells.^[^ ^35^ ^]^ In human, immunohistochemistry analysis of clinical BLCA sections revealed the expression of CK5/6 in MIBC and CK20 in NMIBC, respectively, which was consistent with the findings in mouse study. This study demonstrated that in humans, MIBC originates from basal cells and that the ancestors of papillary NMIBC are potentially superficial intermediate/umbrella cells. Concordance between single cell gene expression and tissue methylation data suggested that the aggressiveness of cancer cells is related to their epigenomic similarity to basal cells. In particular, cancer cells exhibiting high similarity with BsP were the most malignant. This suggests that BLCA cells derived from BsP are naturally malignant and that cancer cells can dedifferentiate into a stem‐like state to a adopt higher malignancy capacity.^[^ ^23^, ^36^ ^]^ Combined with single cell RNA expression analysis, we conclude that human MIBC is derived from a TM4SF1‐positive intermediate progenitor (Figure 8G).

TM4SF1 marks bladder progenitor cells: TM4SF1, a member of the tetraspanin family reported to be a tumor‐specific antigen, promotes proliferation, invasion, EMT, and chemo‐resistance.^[^ ^37^ ^]^ The expression of *TM4SF1* marks stem cells in mesenchymal tissue^[^ ^38^ ^]^ and lung.^[^ ^39^ ^]^ Previously, we identified *TM4SF1* expression as a marker of aggressive high‐grade BLCA.^[^ ^40^ ^]^ *TM4SF1* is co‐expressed with *OCT4*, a well‐known pluripotency marker, in the normal urothelium. This co‐expression is upregulated in MIBC, suggesting that *Tm4sf1* ^+^ marks a progenitor‐like cells in both normal urothelium and BLCA. Here, we identified that *TM4SF1* served as a marker for human and mouse bladder urothelium progenitor cells, suggesting that *TM4SF1* might serve as a general stem cell marker in several tissues with layered epithelium structures.

TPCS is an early tumor initiator directly derived from intermediate progenitor cells: TPCS, which is highly proliferative and transcriptionally plastic, is phenotypically similar to hyperplastic cancer cells reported in other cancer types.^[^ ^3^, ^4^, ^41^ ^]^ In this study, TPCS was demonstrated to be an early way‐point of high‐grade basal BLCA evolution, which is directly derived from an intermediate progenitor cell in the normal urothelium. In both human tissue and mouse organoid models, *Tm4sf1* ^+^ cells were intermediate progenitors derived from bladder stem cells and gave rise to other differentiated cell types. In a mouse OHBBN‐induced high‐grade invasive mouse model, *Tm4sf1* ^+^ cancer cells are the major population that gives rise to further differentiated cancer cells, suggesting that *Tm4sf1* ^+^ cancer cells serve as the multiple lineage progenitors. Transcriptional reprograming of murine and human BsP toward TPCS is highly similar, involving initial de‐differentiation and subsequent EMT. In human BLCA, mitosis age inferred with ChrAcc on ClockDML indicates that TPCS is generated at an early stage. Phylogeny inferred with ChrAcc on ClockDML demonstrated that cells with TPCS epigenotypes are ancestral to other basal cancer cells. These results indicate that TPCS is the first tumor cell derived from TM4SF1‐positive intermediate progenitor BsP.

TPCS may be derived from injury state intermediate progenitor cells: The *Tm4sf1* ^+^ cells in injured tissue in mouse bladder are transcriptionally similar to an intermediate state between normal BsP and cancerous TPCS. Notably, upregulation of TP53 signaling and dedifferentiation is found in *Tm4sf1* ^+^ BsP cells of benign, non‐tumorous differentiating organoids and injured tissues. This is reminiscent of the requirement of TP53 in progenitor cells (“blastema”) during wound healing and regeneration.^[^ ^42^ ^]^ This suggests that cellular dedifferentiation is induced by injury, and TP53 activation is necessary for the induced intermediate BsP progenitor differentiation toward terminal cell fate. Such results partially explain the enrichment of TP53 inactivation in BLCA.

TPCS supports the generation of ITH in BLCA: The basal‐like gene expression profile is variable between TPCS clusters, suggesting lineage plasticity in TPCS epigenotypes. Integrating human scRNA and scATAC data suggests that TPCS epigenotypes bridged the transition between RNA phenotypes. We further showed that TPCS epigenotypes could generate multiple transcriptional phenotypes in human BLCA through multimodal integrative analysis, suggesting that heterogenic human high‐grade basal BLCA evolves through a common TPCS epigenotype state.

In our dataset, all metastatic, squamous differentiation BLCA‐isolated cells were TPCS. In the mouse model dataset, TPCS cancer cells overexpress the luminal markers Xbp1 and Fgfr3 as well as the basal stem cell markers Itga6 and Cd44, the basal marker Egfr, and the EMT‐related marker Cldn4, suggesting that TPCS are of potential to generate multiple lineages. Our findings are most consistent with the scenario in which the TPCS epigenome supports a variety of distinct transcriptional phenotypes (Figure 8G).

Limitations of this study include: 1) a lack of paired single cell ChrAcc and transcriptome profiling, resulting in many of the multimodal joint analyses relying on bioinformatic inference; 2) a lack of in vivo experiments to validate the direct derivation of cancer TPCS from BsP; 3) a lack of in vitro experiments to validate the proposed mechanism; and 4) a lack of detailed analysis on how differential transcription factor activity regulates gene expression, putatively supported by ChIP‐exo and CUT&Tag experiments. These limitations await be addressed by future experiments.

In summary, this study revealed a comprehensive overview of BLCA evolution based on a multi‐omics single cell atlas. Cell‐of‐origin of BLCA corresponds to its clinical behavior. NMIBC and MIBC originate from superficial intermediate/umbrella and basal cells of the urothelium, respectively. In the advanced clinical stage, a progenitor‐like, TM4SF1‐positive cancer cell subpopulation emerges in MIBC driven by BMP signaling and EMT, which contributes to ITH by supporting highly plastic transcriptional phenotypes with its unique epigenome. Distinct molecular events primed the TPCS cells to develop into cancer cells with distinct transcriptional profiles. These data suggested that during BLCA progression, the adaptation of a progenitor/stem cell‐like fate occurred and contributed to ITH in BLCA. The emergence of TPCS supported transcriptional phenotype diversification in tumors and facilitated immune evasion and chemotherapy resistance. This study provides useful insights into BLCA biology and novel clinical therapeutic targets for BLCA therapy.

## 4 Experimental Section

### Human Biospecimens

Basic, clinical, pathological, and follow‐up data records for all patients were collected (Supporting Information Dataset 1) and three pathologists were invited to independently confirm the histological diagnosis. This study was conducted in accordance with the measures of China on the administration of clinical research, the Declaration of Helsinki, and the Institutional Ethic Protocols of Zhongnan Hospital of Wuhan University (ZHWU), Beijing Friendship Hospital, Peking University International Hospital and Hubei Cancer Hospital. The study protocol was approved by the Ethic Institutional Review Board (IRB) at ZHWU (approval number: 2015029 and 2020102). All patients and all relatives of the organ donors provided written informed consent. All study procedures were performed in accordance with the ethical standards of the Institutional Ethics Review Committee. Human sample preservation by the Department of Biological Repositories at ZHWU, the official member of the International Society for Biological and Environmental Repositories (https://irlocator.isber.org/details/60), was approved by the IRB at ZHWU and the China Human Genetic Resources Management Office, Ministry of Science and Technology of China.

Human biospecimens were collected from the clinical practice of the ZHWU, Beijing Friendship Hospital, Peking University International Hospital and Hubei Cancer Hospital. Clinical assessment of bladder cancer was performed according to the EAU 2020 Oncology Guidelines (https://uroweb.org/individual‐guidelines/oncology‐guidelines/). Human peripheral blood was collected in BD EDTA tubes according to the manufacturer's protocol and stored at 4°C for no longer than 8 h before serum separation. Peripheral blood mononuclear cells (PBMCs) were separated with the standard Ficoll protocol. Fresh tumor or normal tissues were collected during surgery and transferred to the laboratory in high glucose, 10% FBS supplemented with DMEM. Tissue samples were resected in PBS prior to single cell dissociation. For methylation sequencing, tissue samples were flash frozen in liquid nitrogen and stored at ‐80 °C.

### Human Public Data

This study used a series of human bladder cancer sequencing data from the NCBI SRA sequencing read archive (https://www.ncbi.nlm.nih.gov/sra) with the accession code PRJNA662018. The TCGA BLCA dataset from UCSC Xena (http://xena.ucsc.edu) was used in the present study. The IMVigor210 dataset was downloaded from http://research‐pub.gene.com/IMvigor210CoreBiologies/packageVersions/. The Encode TFBSs, ChromHMM tracks, CpG islands, repeat region data, and lift‐over chains were collected from the UCSC Genome Browser (http://www.genome.ucsc.edu/). Transcription factor binding information was downloaded from the ReMap database (http://pedagogix‐tagc.univ‐mrs.fr/remap).

### Molecular Biology

*Nucleic acid preparation*: For methylation sequencing or mutation panel capture sequencing, genomic DNA was extracted from tumor or normal tissues using the Qiagen animal tissue DNA extraction kit (Qiagen Cat. #69504), following the manufacturer's instructions. Genomic DNA was extracted from formalin‐fixed paraffin‐embedded tissue sections using the MagPure tissue DNA DF kit (Magen Inc., Cat. #MD5112‐TL‐06). Quality control of the extracted DNA was performed using a Qubit dsDNA HS assay (Thermo Fisher Scientific) and an Agilent 2100 Fragment Analyzer.

### ATAC‐Seq of Tumor Tissues

Flash‐frozen tumor tissue samples (20 mg) were minced using a double‐sized douncer (Sigma, Cat. #D8938) in 1× HB (0.25 M sucrose, 0.06 M KCl, 0.015 M NaCl, 0.005 M MgCl_2_, 0.01 M Tris‐HCl pH 7.5) and digested in 5 mL of trypsin and 40 µL of 5 U µL^−1^ DNase I (Sigma, Cat. #D5025) at 37 °C for 45 mins with two rotations in between to mix the reaction mixture. The digested cells were then neutralized with an equal volume of DMEM (Thermo Fisher, Cat. #11995065) supplemented with 10% FBS (Gibco, Cat. #16000044) and filtered through a 70 µm cell filter (BD Falcon, Cat. #352350). The homogenate was centrifuged at 500 g, and 4 °C for 5 mins. The pelleted cells were then resuspended in 400 µL of 1× HB, washed once, transferred to a 2 mL LoBind tube (Eppendorf), and washed again. The cells were counted using Trypan blue (Solarbio, Beijing, China). After quantification, the cells were incubated with a 30%‐40%‐50% iodixanol (Sigma, Cat. #D1556) gradient and centrifuged at 3000 g, 20 mins at 4 °C. The cell layer at 30%‐40% interface was collected for library preparation. DNA library was prepared (“tagmentation”) using a Tn5 transposase kit (Vazyme, Cat. #TD501) with 1 million cells per reaction, following the manufacturer's instructions. After tagmentation and PCR amplification, the sequencing library was subjected to quality control analysis with SYBR‐green based qPCR using primers for the housekeeping gene (*GAPDH*) promoter and gene desert (chr5: 105187030–105190000) before sequencing.

### Single Stranded DNA Methylation Capture Sequencing

Tissue genomic DNA (200 ng) was bisulfite converted using the EZ‐DNA Methylation‐Gold Kit (Zymo Research, Cat. #D5006) according to the manufacturer's protocol. After conversion, the DNA was subjected to the single‐stranded library preparation protocol Tequila 7N (Euler Technology). In brief, the DNA was end‐repaired using the Klenow fragment (NEB) and tailed with poly‐A homopolymer using terminal deoxynucleotide transferase (Takara, Japan), ligated to a poly‐T overhang adaptor using T4 DNA ligase (Enzymatics), and linearly amplified for 12 cycles using PhusionU (Thermo Fisher Scientific). The amplified linear products were then annealed to a 5’ adaptor with a 7 bp 3’ random nucleotide overhang and PCR‐amplified using adaptor oligos (Sangon, Shanghai, China) and Phusion (Thermo Fisher Scientific), resulting in a library with proper Illumina sequencing adaptor ends ready for next‐generation sequencing (NGS). Hybridization was performed with SeqCap EpiGiant Enrichment Probe (Roche, Cat. #07138911001), oligos and SeqCap wash and binding buffers (Roche) following the manufacturer's protocol. After hybridization, the library was amplified using Phusion for 8 cycles and sequenced on a NovaSeq sequencer (Illumina, CA, USA) to a target of 100 M paired‐end 150 bp reads.

### DNA Mutation Panel Sequencing

DNA was sonicated into ≈250 bp fragments with a Covaris S220 concentrator. Next‐generation sequencing (NGS) libraries were constructed with a single‐stranded DNA ligation protocol. In brief, sonicated DNA was denatured to form a single strand and 3’‐polyA‐tailing was performed with terminal transferase (Enzymatics Ltd., USA, Cat. #P7070). The ligation of a polyT‐extruding adaptor (Sangon Ltd., China) was performed with *E.coli* ligase (Takara Ltd., Japan, Cat. #2161). Linear amplification of the ligated product was performed with adaptor‐specific primers (Sangon Ltd., China) for 12 cycles and the amplified product was annealed and ligated into a 5’‐polyN‐extruding adaptor (Sangon Ltd., China) with T4 ligase (Enzymatics, USA, Cat. #L6030). The ligated product was then amplified with Illumina‐compatible primers (Sangon Ltd., China) for 10 cycles. For panel capture sequencing, the amplified library was captured using either: (1) a custom‐synthesized oncology panel‐consisting of exons, UTRs and structural variant breakpoint‐enriched introns of 538 tumor‐related genes, as well as 1076 SNP loci (Euler Technology Ltd., China), or (2) a custom‐synthesized whole exon panel consisting of exons, UTRs and SNP loci from the human genome (GRCh37), totaling 52.6 Mbp (Euler Technology Ltd., China). Libraries were sequenced to targeting ≈800× (oncology panel) or ≈150× (whole‐exome) on‐target coverage with paired‐end 150 bp read format on an Illumina NovaSeq platform.

### scRNA‐Seq and scATAC‐Seq

Fresh tissues were processed immediately after being obtained from bladder cancer patients. Tissues were cut into tiny pieces (<1 mm diameter) and then subjected to dissociation using collagenase II (Biofrox, Cat. #2275MG100) and 100 µL of DNase (Servicebio, Cat. #1121MG010) at 37 °C for 1 h. After dissociation, the cells were filtered with 40 µm BD filter mesh and subsequently centrifuged at 250 g for 5 mins. The cell pellets were washed in PBS twice, resuspended in 1 mL of ice‐cold RBC lysis buffer and incubated at 4 °C for 10 mins. Then, 10 mL of ice‐cold PBS was added to the tube and subsequently centrifuged at 250 g for 10 mins. After decanting the supernatant, the pellet was resuspended in 5 mL of calcium‐ and magnesium‐ free PBS containing 0.04% weight/volume BSA. The cells were counted using Trypan blue (Solarbio, Beijing, China). For chromatin accessibility sequencing, ≈10^6^ cells were used for nucleus extraction. Nucleus extraction was performed as 10× single cell library preparation according to the manufacturer's protocol. Chrominum Single Cell 3’ V3 kits, 5’ V3 kits (for TCR sequencing), and ATAC V2 kits were used. For RNA sequencing, single cell suspensions were loaded onto a Chromium Single cell Controller Instrument (10× Genomics) to generate single cell gel beads in emulsions (GEMs) targeting ≈8000 cells (3’/5’ RNA). After generation of GEMs, reverse transcription reactions were performed to generate barcoded full‐length cDNA, which was followed by disruption of emulsions using the recovery agent, and then cDNA clean‐up was performed with DynaBeads Myone Silane Beads (Thermo Fisher Scientific). Next, cDNA was amplified by PCR. Subsequently, the amplified cDNA was fragmented, end‐repaired, A‐tailed, and ligated to an index adaptor, and then the library was amplified. To amplify the TCR sequence, a 10× Genomic TCR kit was used according to the manufacturer's protocol. The scRNA libraries were sequenced with the aim of obtaining ≈5000 reads per cell on an Illumina NovaSeq platform with paired‐end 150 bp reads.

For ATAC, tagmentation was performed according to the manufacturer's protocol. After the tagmentation reaction, nuclear suspensions were loaded into a Chromium Single cell Controller Instrument (10× Genomics) targeting ≈10 000 nuclei in one reaction. After the generation of GEMs, PCR was performed to amplify the library. DNA clean‐up was performed with size‐selected XP beads. Libraries were sequenced aiming to have ≈5000 reads per cell via Illumina NovaSeq with paired‐end 50 bp reads.

### Bioinformatics

*Mutation profiling*: The raw sequencing data were mapped using bwa‐mem to the GRCh37 reference genome with default parameters. Germline mutations were called with the Sentieon haplotyper (Sentieon‐Genomics‐201808.05) and annotated with VEP^[^ ^43^ ^]^ (90.1) and SnpSift^[^ ^44^ ^]^ (4.2). For paired tumor‐normal samples, candidate germline variants were filtered with the gnomAD global frequency < 0.001 and in‐house database frequency < 0.001 (out of 20 000 patients). Intersection with variants found in different male members of the pedigree was performed to extract patient‐specific germline mutations. Somatic tumor mutations were called with the Sentieon TNscope (Sentieon‐Genomics‐201808.05) with paired samples or NA12878 (at unpaired cases) and Pisces^[^ ^45^ ^]^ (5.2.9.122), while variants called by both algorithms were passed for filtering. CNVs were called using CNVkit^[^ ^46^ ^]^ with default parameters. B‐allele frequency (BAF) determination was performed for germline and somatic variants. The tumor genome was segmented using BAF and sequencing depth information. Allelic copy numbers were determined for each somatic variant using a hypergeometrical test. Tumor content determined by an in‐house CNV‐based linear regression method was confirmed by hematoxylin and eosin (H&E) staining. A minimal tumor‐cell‐fraction of 5%/2%, a minimal variant read number of 10/3, and a minimal read depth of 500/30 were applied to variants for filtering for panel sequencing or whole‐exon sequencing, respectively. Filtered mutations were annotated with vcfanno and filtered with gnomAD global frequency < 0.001.

### Genomic Region Liftover

For genomic region liftover between UCSC GRCh38 and GRCh37, the R package easyLift (https://github.com/caleblareau/easyLift), the liftover executable from UCSC Kent Utility (https://genome.ucsc.edu/cgi‐bin/hgLiftOver) and the lift‐over synteny chain files from the UCSC Genome Browser were used. The target genome was always GRCh38.

### DNA Methylation Data Processing

Raw bisulfite‐converted DNA methylation sequencing data, either downloaded from NCBI SRA or directly from in‐house sequencing, were processed using fastp (–trim‐front2 20 ‐w 20)^[^ ^47^ ^]^ (https://github.com/OpenGene/fastp) and mapped to the GRCh37+decoy reference genome using BWA‐Meth (https://github.com/brentp/bwa‐meth) using standard parameters. Mapped data were deduplicated and sorted using Sambamba (https://github.com/biod/sambamba) and Samblaster (https://github.com/GregoryFaust/samblaster). CpG‐methylation levels were extracted using the Pile‐O‐Meth (https://github.com/dpryan79/MethylDackel) toolkit. For all libraries, the conversion rate was quality controlled by CHH methylation level > 99%. Basic statistics of the in‐house sequencing library were further quality‐controlled by on‐target rate and on‐target coverage with bedtools (https://github.com/arq5x/bedtools), and duplication rate and mapping rate with Sambamba.

### Differential Methylated Loci (DML) and Region (DMR)

CpG methylation level (beta: defined as reads of C nucleotide over total read coverage on single C bases on both strands on CpG loci) was measured for each CpG loci across the genome as mentioned above using Pile‐O‐Meth. For each loci, beta values from sequencing results were summarized in R (3.6.2) using an in‐house script. DML was defined as: (1) *p* < 0.01 for T‐test between control and case groups (given NMIBC/MIBC or BLCA/normal); (2) beta difference between case and control groups > 0.1. Initial DMR candidates were made by merging within‐100 bp‐apart DML. The average beta of each initial DMR was calculated as the mean beta of all CpGs encompassed in the DMR. This average beta was subjected to T‐test and *p* < 0.01 regions were selected as candidate “seed” DMRs. Segments of methylation difference levels were computed using a circular binary segmentation approach on beta difference case and control groups with DNAcopy^[^ ^46b^ ^]^ (https://github.com/veseshan/DNAcopy). K‐means clustering was performed using R (3.6.2) on the methylation beta difference on each segment, and clusters of segments fully encompassing candidate “seed” DMRs were selected as true DMR candidates.

### ATAC Sequencing Data Preprocessing

Raw paired‐end open chromatin tagmentation (ATAC) sequencing data were mapped to the human reference genome GRCh38 using Bowtie2 (‐k 10 –very‐sensitive ‐X 2000) (https://github.com/BenLangmead/bowtie2). All unmapped reads, non‐uniquely mapped reads, reads with low mapping quality (MAPQ < 20) and PCR duplicates were removed. For in‐house prepared ATAC‐seq data, the data were quality‐controlled by assessing insertion size (using an in‐house R script) and TSS‐enrichment (using an in‐house R script with the GenomicRanges package (https://github.com/Bioconductor/GenomicRanges) measuring the depth ratio at the promoter region (GRCh38 refFlat annotation from UCSC Genome Browser) (0bp of TSS vs. 1kbp +‐ of TSS). A QC‐passed ATAC‐seq library must have TSS enrichment of 6, mapped deduplicated sequencing fragments ≥ 20 M PE reads, PCB1 > 0.9, and PCB2 > 3 (https://www.encodeproject.org/pipelines). Enrichment peaks were determined by intersecting peaks found from MACS2 callpeak (‐f BAMPE)^[^ ^48^ ^]^ (https://github.com/taoliu/MACS) and Genrich (‐r ‐m 1 ‐j) (https://github.com/jsh58/Genrich). Quality control of ATAC‐seq libraries including read length, V‐plot and TSS‐enrichment were performed with custom R scripts and deepTools (https://github.com/deeptools/deepTools). Reliable peaks were identified with IDR (https://www.encodeproject.org/software/idr). Reliable ATAC peaks from different sets of data were converged with 1 bp minimum overlap and extended to the largest width of overlapping peaks. Joining these operation results in a set of non‐overlapping, varied‐width peaks across the genome encompassing all reliable open chromatin regions.

### Measurement of Differences in ATAC‐Seq Peaks

Read coverage of the sequencing library was collected over the repeatable ATAC enrichment peak mentioned above with Sambamba. Differential enrichment was performed with DESeq2^[^ ^49^ ^]^ (https://github.com/mikelove/DESeq2) using standard parameters. For samples with few replicates, we adapted a general linear model approach for estimating differences following the method mentioned in Reilly *et al.* ^[^ ^50^ ^]^

### scRNA‐Seq Data Preprocessing

Loompy‐Kallisto^[^ ^51^ ^]^ was used for to map the RNA data for gene expression analysis. Loom files were read in R by hdf5r (https://cran.r‐project.org/web/packages/hdf5r/index.html) and pre‐processed with Seurat3.0.^[^ ^12b^ ^]^ Quality control was performed for every single sample individually to filter against gene counts, unique molecular identifier (UMI) counts, total reads, and mitochondrial reads. Generally, cells with > 10% mitochondrial reads, or with UMI < 600 or > 5000, or with gene counts > 5000 were filtered prior to subsequent analysis. Such a quality control process might iterate at every subsequent step to ensure the stringency of analysis. Individual samples were processed through the Seurat pipeline. Data first passed the DoubletFinder^[^ ^12c^ ^]^ pipeline with standard parameters to filter against potential doublets. The filtered data were then normalized, and the top 2000 variable genes were identified. Ribosomal proteins, heat shock proteins, and chaperones were intentionally removed from the variable gene list because of the highly inconsistent nature of their behaviors between different tissue types. Gene expression profiles were then scaled and reduced using principal component analysis (PCA) with Seurat. Generally, ≥ 30 PCA components were included in subsequent steps. The FindClusters function was initially performed using a high resolution and then gradually lowered to ensure that the final clusters were less than the PC components. SingleR annotation with human reference and conventional markers were used to categorize the cell clusters. DEGs were identified using Seurat FindAllMarkers function with Wilcoxon test for the cell clusters. Comparison of the identified DEGs with conventional markers was performed to ensure that the clusters contained a relatively pure cell population. Cell‐type‐annotated cells were then separated into different subsets based on their types. In this study, epithelial cells, endothelial cells, fibroblasts, CD8 T cells, CD4 T cells, NK cells, B cells and myeloid cells were considered “pools” for subsequent analysis. After pre‐processing, similar types of cells from different samples were merged and re‐analyzed. Quality control, doublet identification, dimensionality reduction, cluster identification, differentially expressed gene identification, and cell type identification were iteratively performed on these “pure” sets of cells. In such a setting, the cell type composition between samples is relatively homogeneous, and it is usually unnecessary to perform data integration. When sample‐driven variation was evident or in cases when the Dataset contained both 3’ and 5’ scRNA samples, to control against for technical variations, Harmony^[^ ^12a^ ^]^ was applied to datasets with the (vars.to.regress = “chemistry”) option during data scaling. “Contaminant” cells that mis‐segregated into large pools were identified and put back into the unprocessed pool. Iterative processing of cells was done semi‐automatically until all cells were processed. In the end, a total of 133953 cells (61724 epithelial, 5458 endothelial, 15203 fibroblast, 1918 myeloid, 23070 CD4, 12165 CD8, 783 NK and 13632 B cells) were collected for downstream study. Data were visualized with 2D tSNE or UMAP projection.

### Public Mouse Bladder scRNA Data Analysis

The pre‐processed mouse public datasets including normal urothelium (GSE180128 and GSE109774), bladder organoid culture (GSE163029), and OHBBN‐induced BLCA model (GSE146137) were downloaded from NCBI GEO. Raw data of the mouse bladder injury model (SRP301241) were downloaded from NCBI SRA and mapped to the GRCm38 genome using kallisto‐loompy. The mouse data were individually pre‐processed by Seurat, and then integrated by Harmony, with default parameters. Cells were annotated according to gene expression in human counterpart cell. The gene set activity of human TPCS‐specific NMF gene modules was measured for mouse cells using Seurat:Addmodulescore. Differentially expressed genes between Bsc, BsP and TPCS were calculated by Seurat:FindAllMarkers. GSEA analyses were done by fGSEA (v1.22.0) in R (v4.1.0) with reference data in msigdbr (v7.5.1).

### TCR Integration

TCR sequencing data were processed with cellranger‐tcr^[^ ^52^ ^]^ with standard parameters. After barcode matching and TCR de novo assembly, the assembled TCR sequence and clonotypes were extracted. Clones of cells with similar TCRs in the same donor were identified as having exactly similar TCR exon usage combinations as well as similar CD3r nucleotide sequences for all TCRs (alpha and beta chain, if applicable) sequences expressed in the cell. Cells with ≥ 3 TCR sequences were removed. Clonal expansion is defined as a single T cell clone that was observed > 2 times in sequencing data from the donor. Local clonal expansion was defined as the clone being observed > 2 times in one sample and < 2 times in all other samples. Global clonal expansion was defined as the clone being observed > 2 times in ≥ 2 samples. scRNA data were subsequently registered with this TCR information to annotate the clonal identity and clonal expansion status of each T lymphocyte.

### Cell‐Cell Signaling Inference

Cell‐cell signaling inference was done with CellChat^[^ ^53^ ^]^ with cells from normal bladder tissue, bladder cancer tissue, adjacent bladder tissue and metastatic prostate tissue. Cell types with ≤ 20 cells were excluded from the analysis. Signaling probability was extracted from the object.

### Co‐Regulated Transcription Module Inference Using NMF

Non‐negative matrix factorization was performed in R using the iterative NMF (iNMF) internal function from Liger^[^ ^54^ ^]^ with data scaled by sample. After NMF, the weight matrix, variation matrix, and loading matrix were extracted from the object. Prediction of metagene expression in single cell was done with the top 100 contributors of NMF component using Seurat AddModuleScore function.

### Transcription Factor Inference with cisTarget

RcisTarget^[^ ^19b^ ^]^ was used to extract enriched TFBS in the co‐regulated gene modules from NMF (top 100 genes from each component) with the human GRCh38 ‐1000 bp to +500 bp database downloaded from cisTopic (hg38_refseq‐r80_500bp_up_and_100bp_down_tss.mc9nr.feather). After extraction, high confidence co‐regulator TF of regulons with NES > 3.0 were extracted from the data. Specific TF regulating a NMF component was defined as a TF which was found in ≤ 2 components. TF binding affected by methylation was identified as the binding site is derived from the in vitro DNA‐binding activity from the work by Yin *et al.* ^[^ ^19a^ ^]^ Visualization of co‐regulated transcription network is performed with visNetwork (https://cran.r‐project.org/package *= visNetwork*). Matching of TF to cell‐cell signaling event was done manually.

### Developmental Lineage Analysis

Diffusion map of scRNA expression data was computed with destiny.^[^ ^55^ ^]^ RNA velocity analysis of single cell 3’ RNA sequencing data was performed with scVelo^[^ ^56^ ^]^ using Reticulate^[^ ^57^ ^]^ in R (3.6.2) with Python 3. With RNA velocity defined the root cluster, slingshot was performed on diffusion maps to produce minimally spanning tree lineages.

### Cell‐Type Association with Clinical Phenotype in TGCA BLCA Patients

TCGA patient clinical phenotype and RNA‐seq data were downloaded from TCGA XENA. A single cell RNA expression profile was used with above data in analysis of cell‐type association with clinical phenotype, performed by Scissor with parameters ‘alpha = 0.05, family = “cox”’. Association with death was defined as “positive” in Scissor analysis. Hence, “Scissor positive” cell population was defined as associated with malignancy/worse survival, while “Scissor negative” cell population was defined as associated with better prognosis.

### scATAC‐Seq Data Analysis

scATAC‐seq raw reads were mapped with cellranger‐atac.^[^ ^58^ ^]^ The mapped fragment files were then processed by ArchR.^[^ ^17^ ^]^ Quality control, doublet identification, LSI‐based dimensionality reduction, cell clustering, gene expression inference, peakset identification (with MACS2), and marker peak finding were all performed in ArchR. Cells from all samples were pooled in the initial analysis, manually annotated with known markers, and separated into different subsets. The subset data were then subjected to scRNA integration in ArchR using Seurat CCA algorithm, with constraints for integration on large pools of cell types. scATAC signal of tumor epithelial cells (cancer cells) segregate into individuals because of the apparent “chromatin accessibility” change with CNV, which was impossible to be removed even using an integer cap to per‐window reads. Hence, for epithelial cells, CNV analysis was done with the “tile” fragment data with a circular binary segmentation algorithm. These regions were then intersected with CNV profiles of the same tumor from panel sequencing, and resulted “true” copy number varied regions were used as a mask for a second round of LSI‐based dimensionality reduction to perform copy‐number‐invariant clustering of cells. Peaks were called for single cell Dataset using MACS2. Chromatin accessibility on given genomic regions was also calculated in ArchR for scATAC difference analysis. Transcription factor footprinting (activity measurement) was done in ArchR with ChromVar. Co‐accessible regions were calculated with ArchR with ‘addCoAccessibility’ (maxDist = 1000000) and filtered with correlation > 0.1. To visualize scATAC coverage on given DMR regions, grouped single cell coverage files were written by ArchR and profile plots were made with deepTools “plotProfile”.

### Epigenotype

MIBC‐specific DMR and MIBC differential ATAC peaks were collected and merged. The merged “epigenetically change regions” were then used as regions for dimensionality reduction with LSI in ArchR. Epigenotype clusters were then subjected to further dimensionality reduction with a diffusion map, and slingshot lineage inference.

### Multimodal Integration between scRNA and scATAC Non‐Hematopoietic Cells in ArchR

Co‐integration of single cell ATAC data and single cell RNA data was performed by ArchR using ArchR: addGeneIntegrationMatrix with the parameters ‘sampleCellsATAC = 30000,sampleCellsRNA = 30 000’. After integration, the RNA cluster of the closest scRNA cell projected to a scATAC cell was considered as a “match” of the epigenotype of this particular scATAC cell. Such epigenotype match to the RNA cluster (phenotype) was counted in R. The Shannon entropy of the epigenotype‐phenotype match was calculated to infer the transcriptional plasticity of a given epigenotype.

### Joint Trajectory Inference in ArchR for TPCS

Single TPCS and normal epithelial cells according to GLUE integration were selected. Co‐integration of single cell ATAC data and single cell RNA data were performed by ArchR using ArchR: addGeneIntegrationMatrix with parameters ‘sampleCellsATAC = 30 000,sampleCellsRNA = 30 000’. RNA expression profile of scATAC cells were inferred during such process. Pseudotime trajectory inference was conducted in ArchR using the normal epithelial cell cluster as starting point. Motif activity inference and gene‐expression x motif activity correlation were performed in ArchR.

### Alternative Multimodal Integration between scRNA and scATAC Epithelial Cells in GLUE

scATAC peak x cell matrix for epithelial cells from ArchR was exported to the MatrixMarket exchange format in R (v3.6.2). The scRNA gene x cell matrix for epithelial cells from Seurat was written in h5ad format in R (v4.1.3) using MuDataSeurat (v 0.0.0.9000). The exported scATAC and scRNA data were then read into python (v3.8.3) and converted into anndata format (v0.8.0). The scRNA Dataset was preprocessed by scanpy (v1.9.1) with: filter_cells(min_counts = 100, min_cells = 3), highly_variable_genes(n_top_genes = 2000), pca(n_comps = 100), and neighbors(metric = ‘cosine’). The scATAC Dataset was preprocessed by scanpy with: filter_cells(min_counts = 100, min_cells = 3) then scglue (v 0.3.2) by lsi(n_components = 100, n_iter = 15). Guidance graph, model training and model fitting were performed by scglue with default parameters. After model fitting, the scglue‐derived dimensionality reduction products were used to cluster all the single cells from both datasets in scanpy with the leiden algorithm with package leidenalg (v0.8.9).

### Transitional Graph

The frequency of single cells GLUE integration clusters belonging to any scRNA clusters (cell types) or scATAC clusters (epigenotypes) were calculated. Adjacency matrix is defined as the matrix multiplication product from the abovementioned frequency matrix (scRNA+scATAC clusters x GLUE clusters), with elements *w*_ij_ corresponding to the transition probability between single cell clusters *i* and *j*. We consider the adjacency elements with top 1% values as ‘possible connected’ states (≈0.03 in this dataset). The adjacency matrix was then denoised by setting any elements below the top 1% value to zeros. R package igraph (v1.3.1) was used to draw a connectivity graph from the adjacency matrix and derive node‐specific connectivity degrees.

### Cell Cycle Scoring

Single cell RNA cell cycle scoring was performed with Seurat^[^ ^12b^ ^]^ using the “cc.gene.2019” Dataset as the source of G2/M and S‐phase gene sets. For visualization, the G2M.score and S.score of a single cell were added together to suggest the relative “actively cycling” probability.

### Differentiation Potential Assessment

Cellular differentiation potential was assessed by CytoTRACE.^[^ ^26^ ^]^ Briefly, the RNA expression matrix of single cells was extracted from Seurat objects, with all transcripts regardless of their in‐assay‐variability. CytoTRACE analysis was performed on this matrix without downsampling. Per‐cluster median CytoTRACE score was used as an index for the differentiation state of each cell cluster. For the comparison, we selected from the scRNA Dataset all “normal” urothelium cells, and cancer cells that projected to scATAC counterparts. Cancer scRNA cells were labeled by their projected scATAC counterparts.

### SNV Matching in Single Cell Sequencing Data

For mutation expression analysis, a standard cellranger pipeline (https://support.10xgenomics.com/single‐cell‐gene‐expression/software/downloads/latest) was used to produce a bam file from scRNA sequencing data. SNVs were extracted from paired tumor mutation panel capture sequencing results and lifted‐over to hg38 using easyLiftOver based on LiftOver. Only somatic mutations with > 5% VAF were included in the analysis. Vartrix (https://github.com/10XGenomics/vartrix) was used to build a cell x mutation matrix.

### Metagene Activity in Bulk RNA Sequencing Data

Metagene was defined as the top 100 genes that contributed to an NMF component. Raw read count matrix of bulk BLCA tumor tissue RNA sequencing data was downloaded from the UCSC Xena and IMVigor210 sources. Limma^[^ ^59^ ^]^ was used to normalize the RNA‐seq count matrix, and GSVA^[^ ^60^ ^]^ was used to score metagene activity. Patients/samples were classified as “high”/ “low” with respect to metagene activity using a 2‐mode Gaussian distribution fitting with mixtools.^[^ ^61^ ^]^

### Survival Analysis

Sample‐centered clinical data were downloaded from the UCSC Xena and IMVigor210 sources. Survival plots and analyses were performed with the R package survplot (http://www.cbs.dtu.dk/~eklund/survplot/).

### Mitotic Age Inference Analysis and Phylogenetic Tree Construction Using ClockDML

The mitotic age of single cells in the scATAC dataset was inferred by EpiTrace^[^ ^25^ ^]^ (https://github.com/MagpiePKU/EpiTrace, v0.0.0.900) following the recommended protocol without tuning the parameters. The EpiTraceAge result from mitosis‐related ClockDML was considered as the mitotic age. Chromatin accessibility on mitosis‐related ClockDML was used for construction of an epithelial cell phylogenetic tree, also using the EpiTrace package.

### Statistical Analysis

No statistical methods were used to determine the sample size used in the study. Methods for pre‐processing of the data including the exclusion criteria of low‐quality cells, mutations or genes/loci were stated in the relevant Methods subsections, namely “Mutation profiling”, “DNA methylation data processing”, “ATAC sequencing data preprocessing”, “scRNA‐seq data preprocessing”, and “scATAC‐seq data analysis”. Methods for normalization and transformation of the data were stated in the relevant Methods subsections, namely “ATAC sequencing data preprocessing”, “scRNA‐seq data preprocessing”, “scATAC‐seq data analysis”, and “Public mouse bladder scRNA data analysis”. Evaluation of outliers has not been performed and no statistical outlier datapoints have been dropped. For data presentation, categorized (grouped) numerical data were always presented as box‐whisker's plot showing mean +/‐ standard deviation, usually overlaid with a width‐normalized violin plot to show the detail distribution of data. Individual data points were always shown when the data points were less than 20. For single cell categories, individual data points were not shown to avoid cluttering of the visualization. Heatmaps and dotplots of gene expression or transcription factor activities were shown with scaled (Z‐normalized) values from blue (lowest) to red (highest) Z‐scores, and the maximal Z‐score were always noted in the accompanying color bar. Heatmaps of prevalence were shown with non‐scaled raw data values. Between‐group statistics were performed with T‐test (two‐sided) or Wilcoxon Rank Test if the T‐test was not applicable due to the distribution, which was justified by KS test. P‐value numbers were shown for each statistical comparison result. Multiple testing adjustment, where applicable, were performed using BH method. Survival analysis was done by the Cox method. Detailed statistical methods were described in the figure legends or accompanying text. Number (n) of tested samples were shown in the figures or legends, where applicable. The overall number of tested single cells for some specific analysis were stated in the Methods section. All statistical analyses in this study were performed using R (3.6.2 or 4.1.3) (http://CRAN.R‐project.org).

## Conflict of Interest

W.J., K.W., L.C., L.S., X. Chen, D.L. and Y.Z. are employees of Euler Technology, Beijing, China. X.W., Y.X., Y.Z., K.Q., M.P., L.J. and G.W. are co‐inventors in a patent application (202210391403.8) for an in vitro diagnostic kit of bladder cancer co‐filed by Zhongnan Hospital of Wuhan University, Kunming Institute of Zoology of Chinese Academic of Sciences, and Euler Technology. All other authors declare no conflict of interest.

## Author Contributions

Y.X., W.J., K.Q., L.J., and G.W. contributed equally to this work. Y.X., Y.Z. and X.W. conceived and designed the study. Y.X., W.J., K.Q., K.W. and Y.Z. contributed to the methodology; W.J., K.W., L.S., F.C. and Y.Z. performed the computations and formal analyses; Y.X., W.J., K.Q., L.J., G.W., L.C., X. Chen, L.S., M.P. and Y.Z. performed the experiments and investigations; K.Q., L.J., G.W., R.C., Z.X., J.L., F.Y., D.L., H.C., W.Z., D.C., Y.T., C.J., Y.L., X.H., H.M. and X.W. contributed to the collection and obtained the study resources. Y.X., W.J., K.Q., L.J., G.W., R.C., Y.W., X. Cao, M.P., Y.Z. and X.W. wrote the original draft, review, and editing with the help of all the authors; Y.X., W.J., K.Q., L.J., G.W., Y.Z. and X.W. wrote critical reviews and commentaries during the revisions. All authors reviewed the manuscript.

## Supporting information

Supporting Information

Supporting Information

## Data Availability

Data availability All data, including raw data and clinical information, have been submitted to the Genome Sequence Archive for Human (http://bigd.big.ac.cn/gsa‐human/) at the BIG Data Center, Beijing Institute of Genomics, Chinese Academy of Sciences, under the accession number HRA001225. The raw sequencing data and clinical information are unique to an individual and require controlled access. The deposited and publicly available data are compliant with the regulations of the China Human Genetic Resources Management Office, Ministry of Science and Technology of China. The original code used during the study were provided at Github: https://github.com/MagpiePKU/Bladder_TPCS_Paper.
