# Supplementary material for: Integrative Single Cell Atlas Revealed Intratumoral Heterogeneity Generation from an Adaptive Epigenetic Cell State in Human Bladder Urothelial Carcinoma
Source: Adv Sci (Weinh). 2024 Apr 6;11(24):2308438. doi: 10.1002/advs.202308438 (PMC11200000; doi:10.1002/advs.202308438)
Supplement: Supplementary file 1 — Supporting Information [file ADVS-11-2308438-s002.pdf]

ADVANCED SCIENCE
Open Access

Supporting Information

for *Adv. Sci.*, DOI 10.1002/advs.202308438

Integrative Single Cell Atlas Revealed Intratumoral Heterogeneity Generation from an Adaptive Epigenetic Cell State in Human Bladder Urothelial Carcinoma

Yu Xiao, Wan Jin, Kaiyu Qian, Lingao Ju, Gang Wang, Kai Wu, Rui Cao, Luyuan Chang, Zilin Xu, Jun Luo, Liuying Shan, Fang Yu, Xintong Chen, Dongmei Liu, Hong Cao, Yejinpeng Wang, Xinyue Cao, Wei Zhou, Diansheng Cui, Ye Tian, Chundong Ji, Yongwen Luo, Xin Hong, Fangjin Chen, Minsheng Peng, Yi Zhang and Xinghuan Wang**

Supplementary Information

Integrative single cell atlas revealed intratumoral heterogeneity generation from an adaptive epigenetic cell state in human bladder urothelial carcinoma

Supplementary Notes: Pages 2-4

Figures S1-S18: Pages 5-30

Description of Supplementary Datasets: Pages 31-33

Supplementary Notes

Normal epithelial cells of the uroepithelium

This study characterized 1,163 normal epithelial cells from healthy organ donors in detail (Figure S9A). These cells were classified into basal (Bs1/2/3), intermediate (Im1/2/3), umbrella (Um), and urothelium stem/progenitor (BsP; whose numbers were fewer) cells based on RNA expression (Figure S9A). The expression levels of *KRT5* and *ITGA6* (CD49f) were upregulated in basal cells. The distinctive features of Bs2/3 cells relative to BsP/Bs1 cells were the upregulated levels of *PCSK5*, *SGK1*, *HES1*, *PRKCZ*, *HOXA3*, and *HOXA5* and the lack of *HBEGF* or *GPX4* expression (Figure S9C). BsP cells exhibited upregulated expression of proliferation-related genes, such as *MKI67* but did not exhibit *HES4* expression. Additionally, the expression of *Tm4sf1* was detected in BsP cells. Meanwhile, the expression levels of *GATA3*, uroplakin-encoding genes (*UPK2*/*UPK3A*/*UPK3B*), and *PIGR* were upregulated in intermediate/umbrella cells (Figure S9C). CXCL6 was a marker of the umbrella cells. Diffusion map inference of the developmental trajectory revealed that the basal progenitor cell evolves into the following three branches: Bs1, Bs2/Bs3, and Im1/2/3/Um (Figure S9B). This is consistent with the findings of previous studies [1]. Consistent with the results of trajectory analysis, analysis with the CellCycleScoring algorithm revealed that the proliferation of BsP cells was significantly higher than that of other urothelium cells, suggesting that BsP cells are the proliferating progenitors for uroepithelium (Figure S9D).

Notch signaling is reported to be essential for urothelium differentiation [2]. In the normal urothelium, the major source of Notch signaling may be from the stromal compartment. The expression levels of the Notch ligands *DLL1* and *JAG1* in fibroblasts were markedly higher than those in epithelial cells (Figure S9C and Figure S18). As Notch signaling is contact dependent, this expression pattern indicates that Notch signaling is spatially confined to the basal cell layer, which is close to the stromal cells. The specific expression of the Notch downstream targets *HES1* and *HES4* and the upregulation of the Notch receptors *NOTCH1* and *NOTCH3* in basal cells suggest that Notch activity is upregulated in basal cells (Figure S9C). Furthermore, cell-cell signaling analysis revealed mutual Notch signaling between basal and intermediate/umbrella cells (Figure S9E). These results suggest that the normal urothelium is derived from basal cells under the control of Notch.

DNA hypomethylation accompanies TPCS generation

Since epigenotypes are defined as cells with similar ChrAcc profiles on tumor-associated DMR, we first analyzed how these profiles change between epigenotypes. Two distinct patterns of ChrAcc shift were identified related to tumor-associated DMRs (Figure S10A). A subset of DMRs, mostly hypermethylated in BLCA (hyperDMR), was exclusively "open" and accessible in stromal cells but remained closed in all epithelial cells irrespective of their malignancy status (Figure S10A, B). Meanwhile, a second class of DMRs, mostly hypomethylated in BLCA (hypoDMR), exhibited an increased accessibility in cancer cells compared with that in both healthy epithelial and stromal cells (Figure S10A, C). The TPCS-related genes *KRT6A*/*KRT6B* and *KRT17* were associated with DNA hypomethylation and exhibited increased ChrAcc in cancer cells (Figure S10A).

As DNAm is typically accompanied by an inaccessible chromatin state, the findings of this study suggest that DNAm levels at many hyperDMRs remain unchanged during bladder cancer oncogenesis. Consequently, the apparent DNA hypermethylation of most of these regions in tumor tissue results from cancer clonal expansion. Conversely, active DNA demethylation occurs at hypoDMRs during BLCA oncogenesis. Such a model implies that epigenotype evolution is mainly accompanied by changes in the ChrAcc and DNAm states on hypoDMRs. On the other hand, ChrAcc and DNAm states on hyperDMRs can serve as a tool for distinguishing cancer cells originating from different cell types, as they are more likely to remain constant during tumorigenesis. The ChrAcc of hyperDMRs changed little between cancer and normal epithelial cells, whereas hypoDMRs were more accessible in cancer cells (Figure S10D). This result is consistent with that of previous studies, which reported predominant DNA hypomethylation in BLCA [3].

DNA hypermethylation marks the cell-of-origin in BLCA

We performed unsupervised hierarchical clustering analysis on the DNAm profiles of hyperDMR in BLCA. Interestingly, the papillary, low-grade, non-invasive Ta tumors and the high-grade invasive T1-T4 tumors, which include all muscle-invasive bladder cancer (MIBC) samples, could be classified by DNAm levels on a core subset of hyperDMR (Figure S10E). The correlation of DNAm levels at core hyperDMRs with their adjacent gene expression levels suggests that different

BLCAs may arise from distinct cell-of-origin. In particular, hyperDMRs specific to NMIBC were detected adjacent to genes whose expression levels in basal cells were higher than those in intermediate cells (*GPX4*/*PRKCZ*/*CD44*) (Figure S9C and Figure S10E). Conversely, hyperDMRs specific to high-grade tumors were associated with genes upregulated in intermediate cells, such as *YWHAZ* and *GATA3* (Figure S9C and Figure S10E). This study prospectively sampled BLCA tumors before the assay. Hence, the stage of the tumor may be directly associated with its aggressiveness. Tumors with a high malignant potential may be highly likely to be sampled at an advanced clinical stage. A subgroup of T3/T4 MIBC cases exhibited hypermethylation in all core hyperDMRs and exhibited a correlation with the BsP origin based on adjacent gene expression (Figure S9C and Figure S10E). This suggests that the most aggressive BLCA tumors directly originate from BsP or that aggressive tumors transform to adopt a BsP-like epigenome (Figure S10E). Together, our results suggest that human BLCA has a heterogeneous origin, with the papillary low-grade tumors arising from superficial urothelium cells and high-grade tumors developing from basal cells.

References

[1] S. A. Colopy, D. E. Bjorling, W. A. Mulligan, W. Bushman, *Dev Dyn* **2014**, *243* (8), 988, https://doi.org/10.1002/dvdy.24143.

[2] C. P. Santos, E. Lapi, J. Martinez de Villarreal, L. Alvaro-Espinosa, A. Fernandez-Barral, A. Barbachano, O. Dominguez, A. M. Laughney, D. Megias, A. Munoz, F. X. Real, *Nat Commun* **2019**, *10* (1), 4407, https://doi.org/10.1038/s41467-019-12307-1.

[3] S. Saghafinia, M. Mina, N. Riggi, D. Hanahan, G. Ciriello, *Cell Rep* **2018**, *25* (4), 1066, https://doi.org/10.1016/j.celrep.2018.09.082.

Figures S1-S18

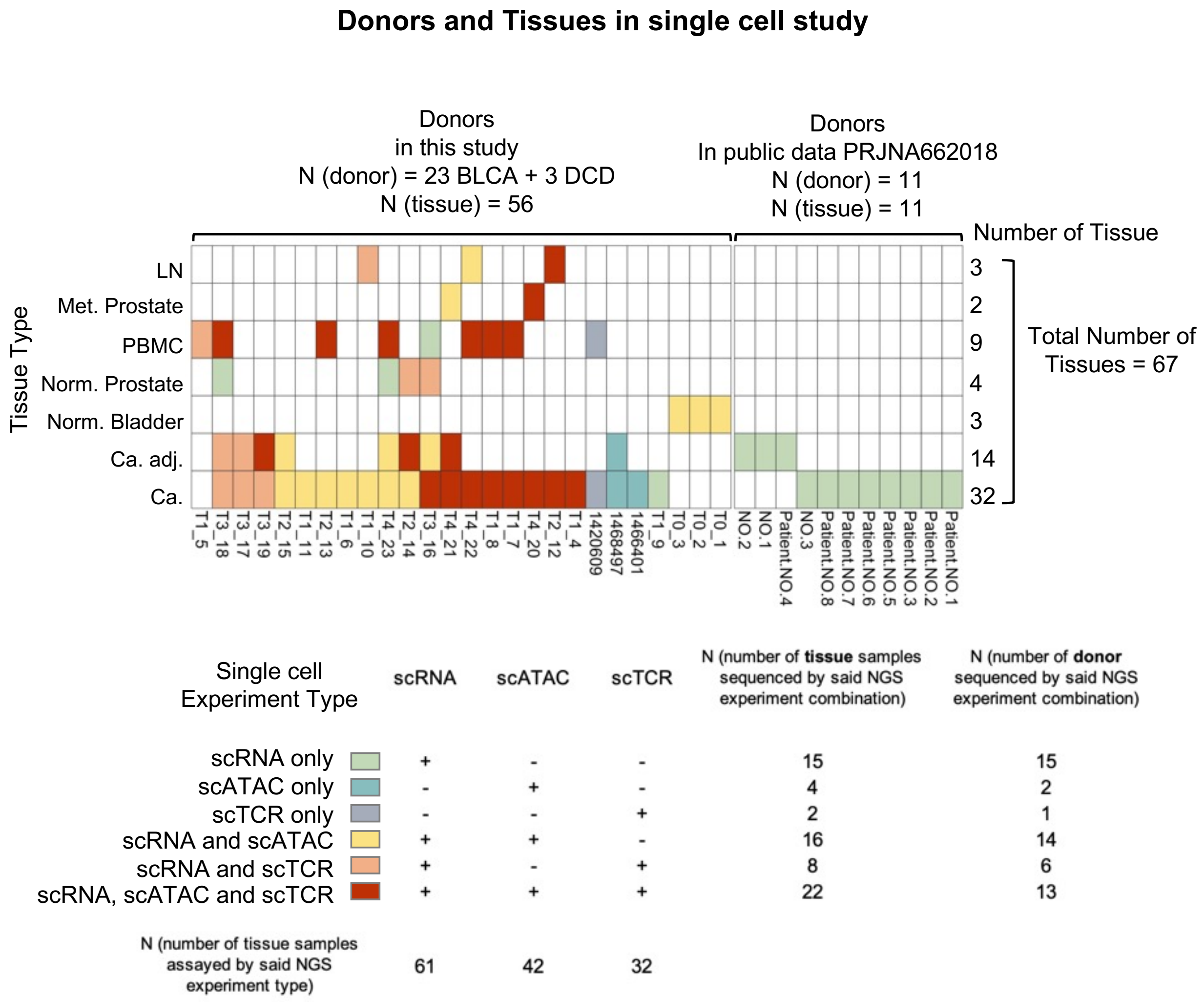

Figure S1. Donors and tissues in single-cell study, related to Figure 1.

The donor ID and tissue type are displayed on the X and Y axes, respectively, with each tile colored according to the single-cell experiment type, as indicated in the lower panel. The number of tissues for each type is listed on the right, and the number of donors is listed at the top. The single-cell study encompassed a total of 37 donors, which included 26 donors in this study and 11 donors from PRJNA662018 dataset. This analysis featured 67 tissue samples across various types. Specifically, from PRJNA662018 dataset, we analyzed 11 tissues, 7 cancer (Ca.) and 4 cancer adjacent (Ca. adj.) tissues, while the remaining samples were directly involved into this study. The tissue types analyzed in single-cell study comprised cancer tissues (Ca.), cancer adjacent tissues (Ca. adj.), normal bladder tissues from DCD donors (Norm. Bladder), normal prostate tissues

(Norm. Prostate), peripheral blood mononuclear cells (PBMC), metastatic prostate tissues (Met. Prostate), and lymph nodes (LN). For each tissue sample, we either conducted a single-cell assay (scRNA, scATAC, scTCR) or a combination of these assays. The specific count of each type of bulk assay performed is detailed in the lower panel.

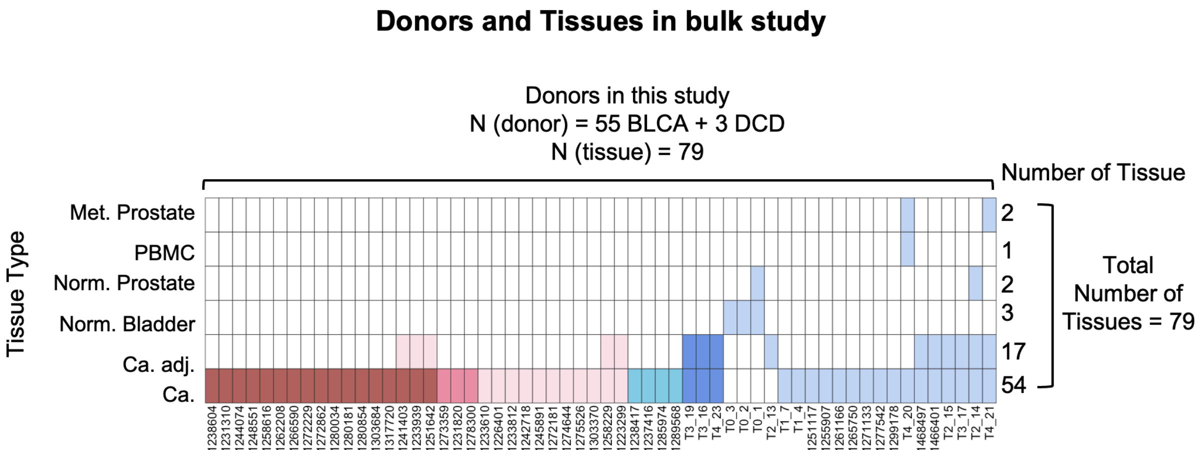

Bulk Tissue Experiment Type	Mutation analysis	DNA Methylation	Bulk ATAC	N (number of **tissue** samples sequenced by said NGS experiment combination)	N (number of **donor** sequenced by said NGS experiment combination)
Mutation analysis only	+	-	-	33	20
DNA Methylation only	-	+	-	6	3
Bulk ATAC only	-	-	+	4	4
Mutation and DNA Methylation	+	+	-	16	14
Mutation and bulk ATAC	+	-	+	3	3
Mutation, DNA Methylation and bulk ATAC	+	+	+	17	17
N (number of tissue samples assayed by said NGS experiment type)	69	39	24		

Figure S2. Donors and tissues in bulk study, related to Figure 1.

The donor ID and tissue type are displayed on the X and Y axes, respectively, with each tile colored according to the bulk tissue experiment type, as indicated in the lower panel. The number of tissues for each type is listed on the right, and the number of donors is listed at the top. The bulk study encompassed a total of 79 tissues from 58 donors in this study. Bulk analysis examined a variety of tissue types: cancer tissues (Ca.), cancer adjacent tissues (Ca. adj.), normal bladder tissues from DCD donors (Norm. Bladder), normal prostate tissues (Norm. Prostate), peripheral blood mononuclear cells (PBMC) and metastatic prostate tissues (Met. Prostate). Each tissue sample was underwent either one bulk assay (Mutation analysis, bulk DNA mehtylation, bulk ATAC) or a combination of these assays. The specific count of each type of bulk assay performed is detailed in the lower panel.

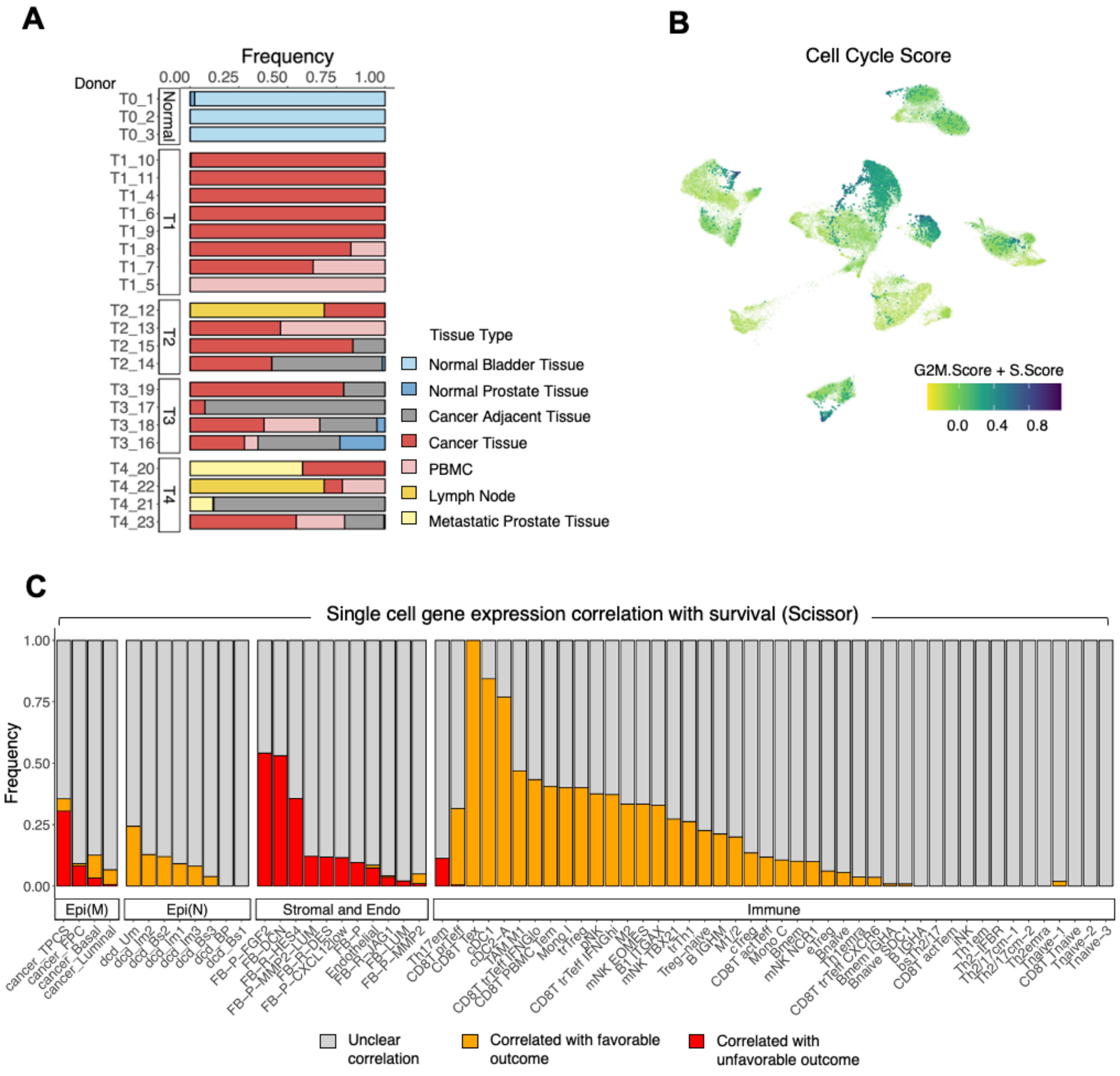

Figure S3. scRNA cell features, related to Figure 1.

(**A**) Tissue-of-origin of scRNA cells for each donor. Single or multiple samples could be taken from a single donor. Donor/patient ID on Y axis was designed by combing the tumor stage of a donor with a sequential identification number. **(B)** Cell cycle score (G2M.Score + S.Score) of scRNA cells visualized on UMAP by color. **(C)** Single cell gene expression correlation with survival (Scissor) analysis of scRNA cell types. Red: unfavorable clinical outcome. Orange: favorable clinical outcome. Gray: unclear correlation. Epithelial cells are classified as cancer (EpiM) or normal (EpiN).

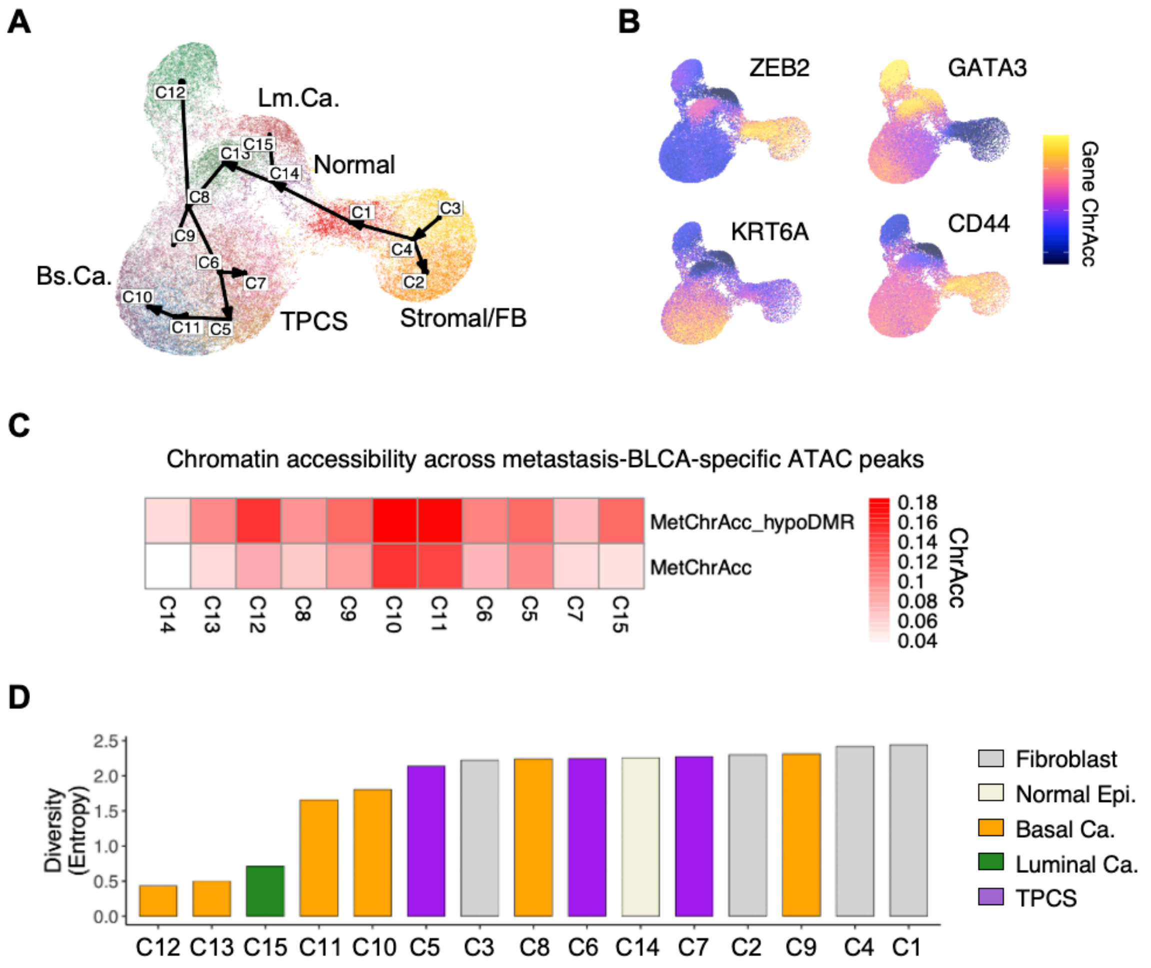

Figure S4. Epigenotype of scATAC cells, related to Figure 3.

(A) UMAP projection of non-hematopoietic BLCA scATAC cells. Dimensionality reduction was performed on chromatin accessibility on CNV-free DMR then clustered via LSI. Single cell cluster (epigenotype) centers are labeled on the map. Monocle-inferred trajectory was shown on the map. **(B)** Chromatin accessibility-inferred gene expression score of marker genes (ZEB2: fibroblast/stromal; GATA3: normal epithelial/luminal cancer; KRT6A: basal; CD44: fibroblast/basal cancer). **(C)** Clusterwise chromatin accessibility on metastatic BLCA-specific ATAC peaks. MetChrAcc: all metastatic-BLCA-specific ATAC sites; MetChrAcc-hypoDMR: metastatic-BLCA-specific ATAC sites that showed DNA hypomethylation. **(D)** Cluster-wise donor diversity of each epigenotype. TPCS epigenotypes showed the highest overall donor diversity.

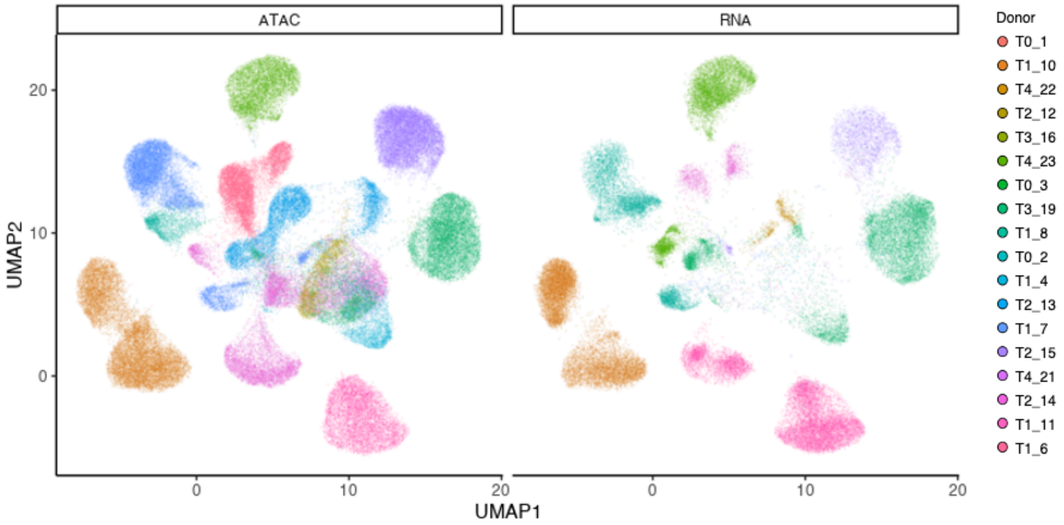

Figure S5. Donor origin of single cells in the multimodal integration dataset, related to Figure 3.

(Left) UMAP projection of scATAC cells or **(Right)** scRNA cells in the multimodal integration dataset. Cells are colored by their donor origin. scATAC and scRNA cells from similar donor are spatially close to each other, indicating accuracy of the multimodal integration. Donor/patient ID was designed by combing the tumor stage of a donor with a sequential identification number.

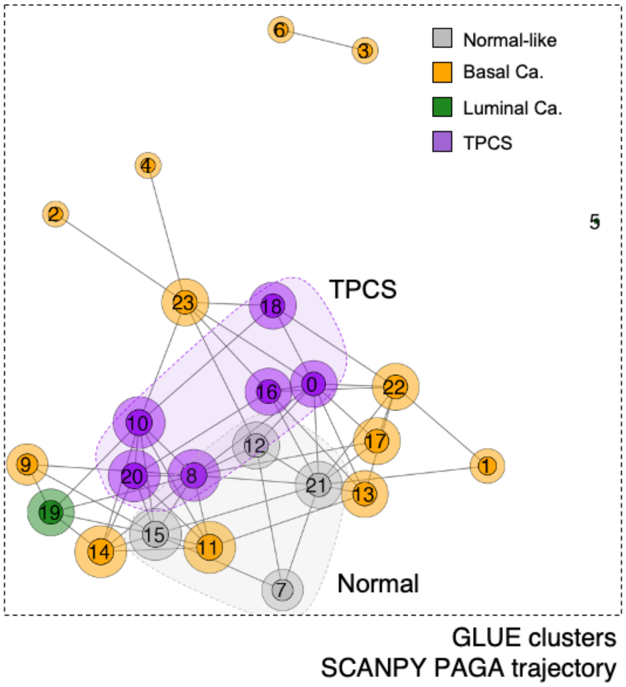

Figure S6. Developmental trajectory between single cell clusters in the multimodal integration dataset, related to Figure 3.

Single cells, irrespective of their origin (scATAC or scRNA), are clustered by multimodal integration (GLUE). Cell types of these single cell clusters are annotated by the majority of scRNA/scATAC cells. Developmental trajectory between these single cell clusters is inferred by PAGA.

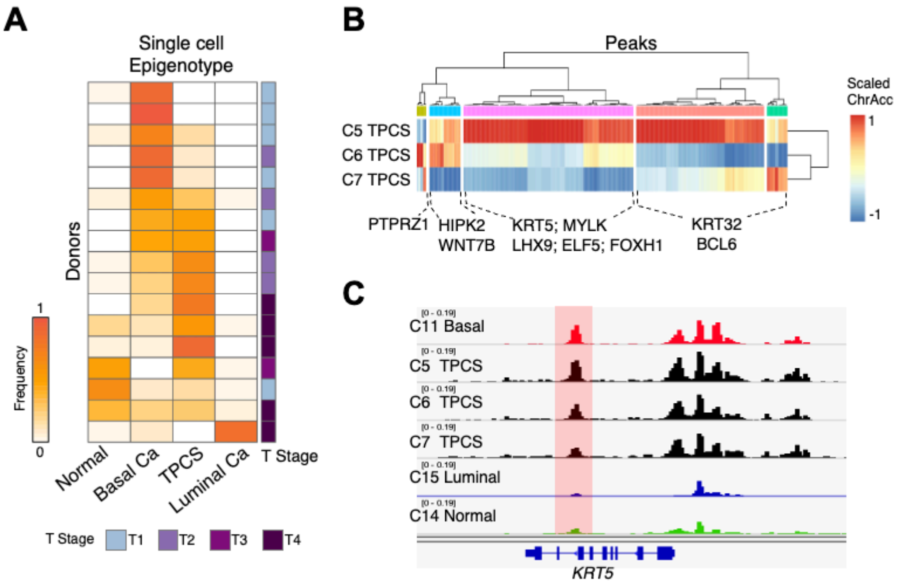

Figure S7. Chromatin accessibility heterogeneity within TPCS epigenotypes, related to Figure 3.

(A) Relative prevalence of scATAC cell types in cancer tissues from different donors. Clinical stages (T) are labeled for each donor on the right color bar. **(B)** Differentially accessible region (peaks) between TPCS epigenotypes. **(C)** Chromatin accessibility on a differentially accessible region in KRT5 intron for normal (C14), luminal cancer (C15), basal cancer (C11), and TPCS (C5, C6, C7) cells.

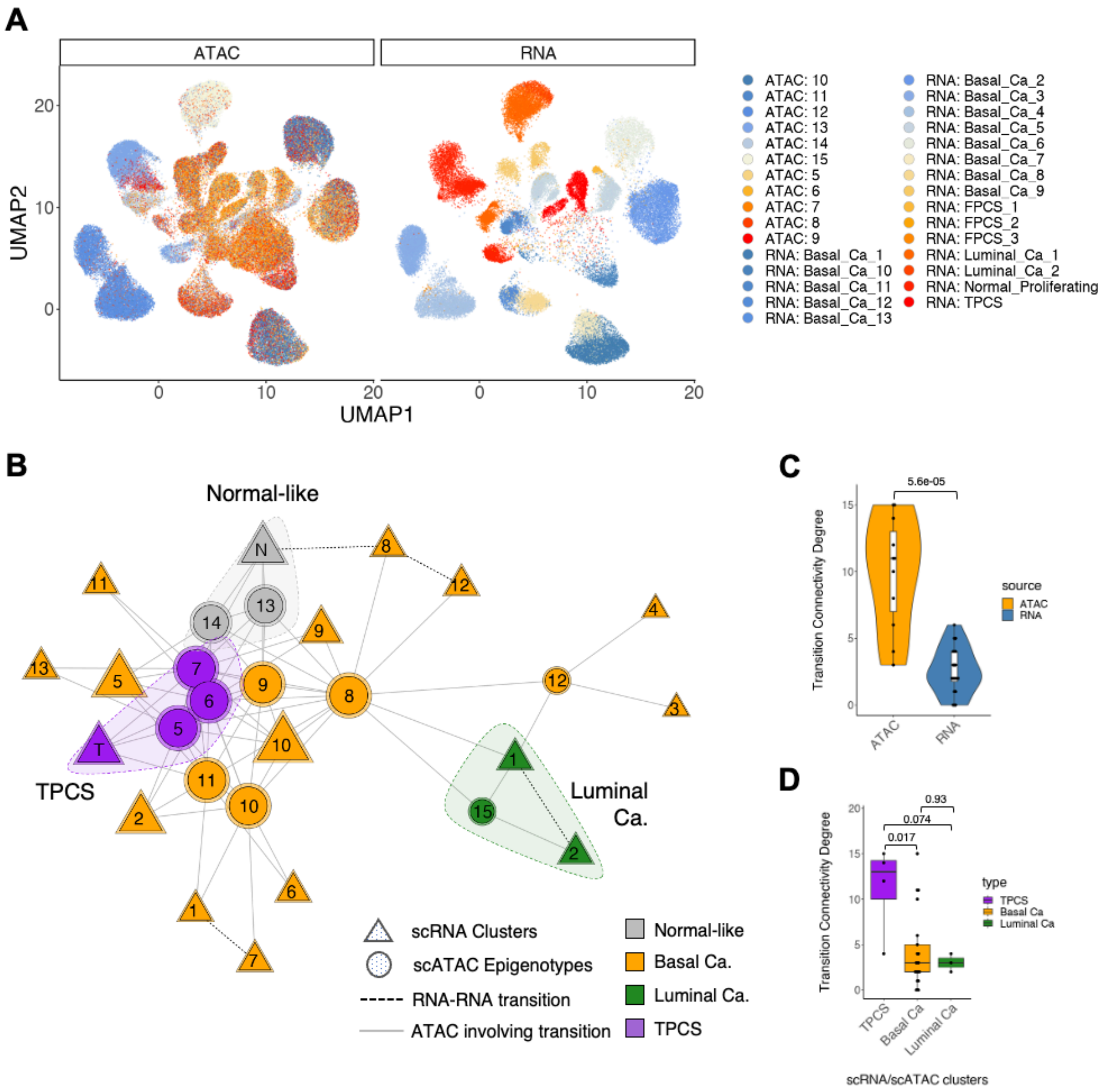

Figure S8. Epigenotype bridges transcriptional transition in BLCA, related to Figure 3.

(A) UMAP of scATAC epigenotypes (left) or scRNA cell types (right) in the multimodal integration dataset. Whilst scRNA cell types are clearly segregated from each other, scATAC epigenotypes are mixed, suggesting phenotypic variability within epigenotypes. **(B)** Transition graph between scRNA and scATAC clusters. Few direct RNA-RNA transitions (black dashed line) between scRNA clusters (triangle) are found. Most transitions are bridged by scATAC epigenotypes (circles). **(C)** Degree of connectivity for each scATAC epigenotype or scRNA cell type. **(D)** Degree of connectivity of different single cell clusters (TPCS, basal cancer, or luminal cancer). *P*-value: wilcox test.

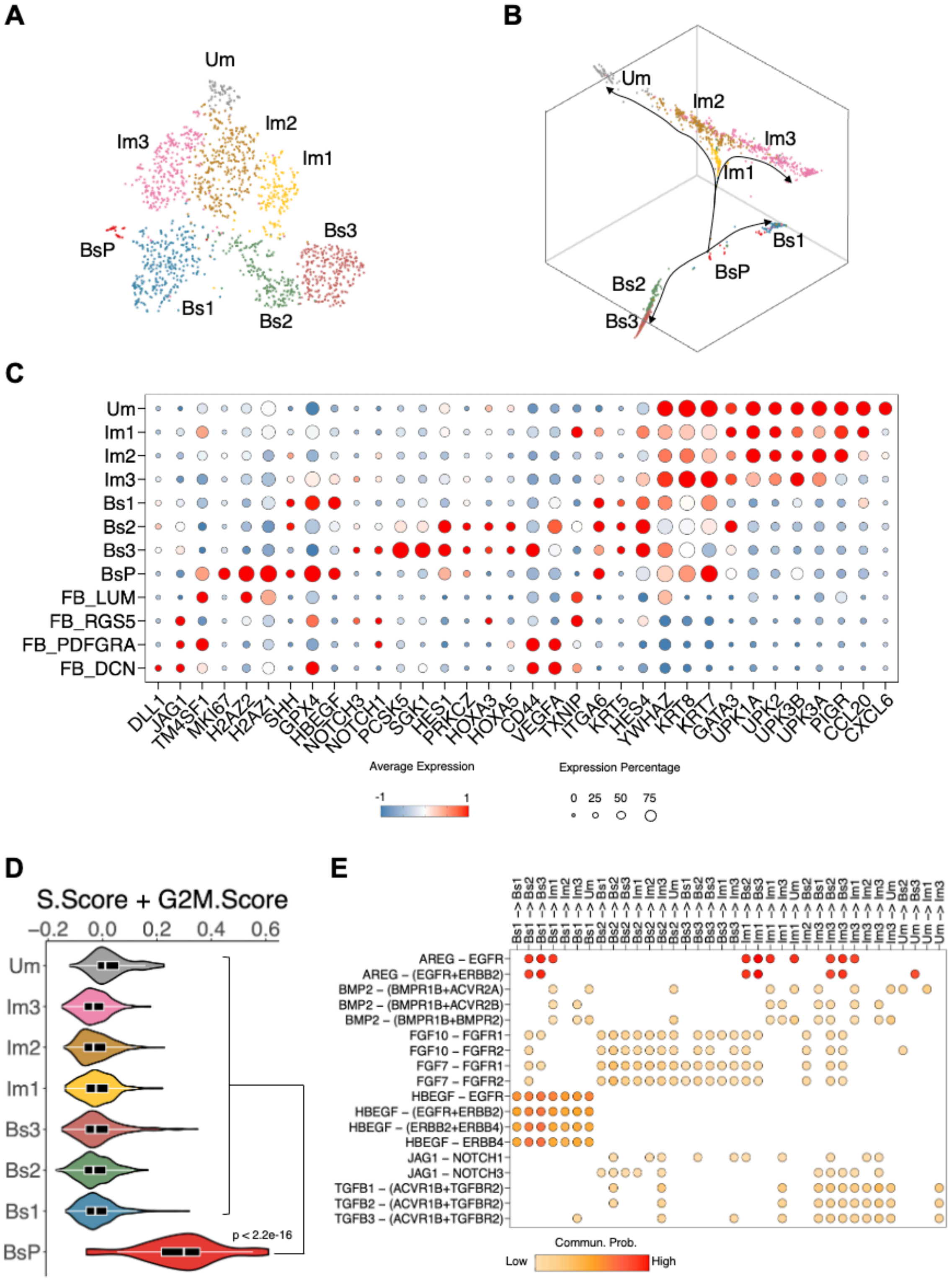

Figure S9. Basal cells generate the normal urothelium, related to Supplementary Notes.

(A) TSNE-projection of normal bladder urothelium epithelial cells from the 3 healthy organ donors. BsP: basal progenitor cell ($SHH^+/CD44^+/TM4SF1^+$); Bs1: basal cell 1 ($IGFBP2^+/KRT17^+/PTGS2^+$); Bs2: basal cell 2 ($HES1^+/ELF3^+$); Bs3: basal cell 3 ($HES1^+/PCSK5^+$); Im1: intermediate cell 1 ($TXNIP^+$); Im2: intermediate cell 2 ($MMP7^+$); Im3: intermediate cell 3 ($GDF15^+/KRT13^+$); Um: umbrella cell ($CD24^+/KRT18^+$). **(B)** 3D diffusion map projection of the single cells and principal curve analysis result showing that the basal progenitor cell evolves into three lineages (Bs1, Bs2/Bs3, and Im1/2/3->Um). **(C)** Single cell gene expression of epithelial and fibroblast cells in the normal urothelium. *KRT5* and *CD44* are expressed specifically in basal cells. The uroplakins *UPK1A*, *UPK2*, *UPK3A*, *UPK3B* are specifically expressed in intermediate and umbrella cells. Notch signaling pathway ligand/receptors, *HES1* and *VEGFA*, are only positive in BsP/Bs1/Bs2. Umbrella cell is identified by *CXCL6* expression. Whilst Notch downstream effector *HES1*/*HES4* are upregulated in basal cells, main source of Notch ligand DLL1/JAG1 comes from the underlying endothelial/fibroblast population. **(D)** Cell cycle score of each cell cluster colored as in (A), suggesting BsP is a major proliferating population. **(E)** Cell-cell signaling probability between epithelial cells suggesting Bs1 provides HBEGF to all other cells, Bs2/Bs3 providing FGF, and intermediate cells contribute BMP/TGFβ ligands. *P*-value: wilcox test.

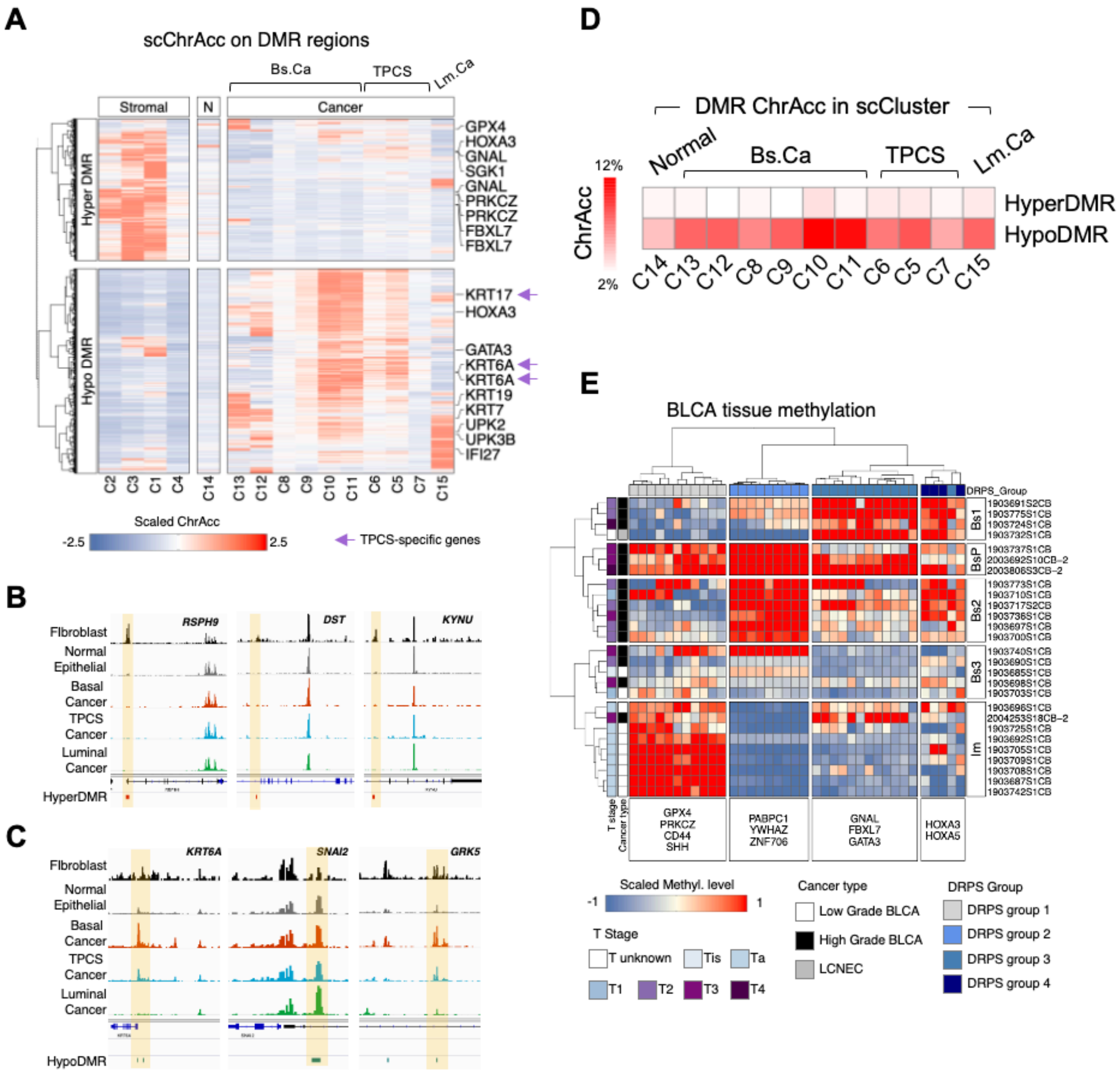

Figure S10. DNA hypomethylation and epigenome reprograming drives BLCA evolution, related to Supplementary Notes.

(A) Single cell chromatin accessibility of non-hematopoietic cells on MIBC-specific differentially methylated regions (DMR). DMR are classified as hypomethylated or hypermethylated in bladder cancer. Single cells are classified by epigenotypes into stromal (2/3/1/4), normal epithelial (14), or cancer (5-13 and 15). MIBC hypermethylated DMR (hyperDMR) chromatin are only open in stromal cells and their chromatin accessibility does not change between normal epithelial cells and cancer**.** MIBC hypomethylated DMR (hypoDMR) chromatin are closed in not only stromal but also normal epithelial cells, and their chromatin accessibility varied between different cancer cells.

DMR adjacent to TPCS-specific genes are labelled by purple arrows. **(B)** Detailed examples of chromatin accessibility around hyperDMR. **(C)** Detailed examples of chromatin accessibility around hypoDMR. **(D)** Overall chromatin accessibility in hypoDMR and hyperDMR across the epithelial epigenotypes. **(E)** Unsupervised hierarchical clustering of DMR methylation level (Z-scaled) of BLCA tissue samples on selected hypermethylated DMR. Samples were labeled by invasiveness (Invasive) and clinical stage (T). DMR are clustered into four groups (DRPS Group). Low-grade, non-invasive papillary BLCA are clearly separated from high-grade, invasive BLCA by hypermethylation in DRPS group 1 DMR, which is adjacent to GPX4/PRKCZ/SHH/CD44 genes. High-grade BLCA could be further classified into 4 clusters by DMR methylation level in DRPS groups 2, 3 and 4.

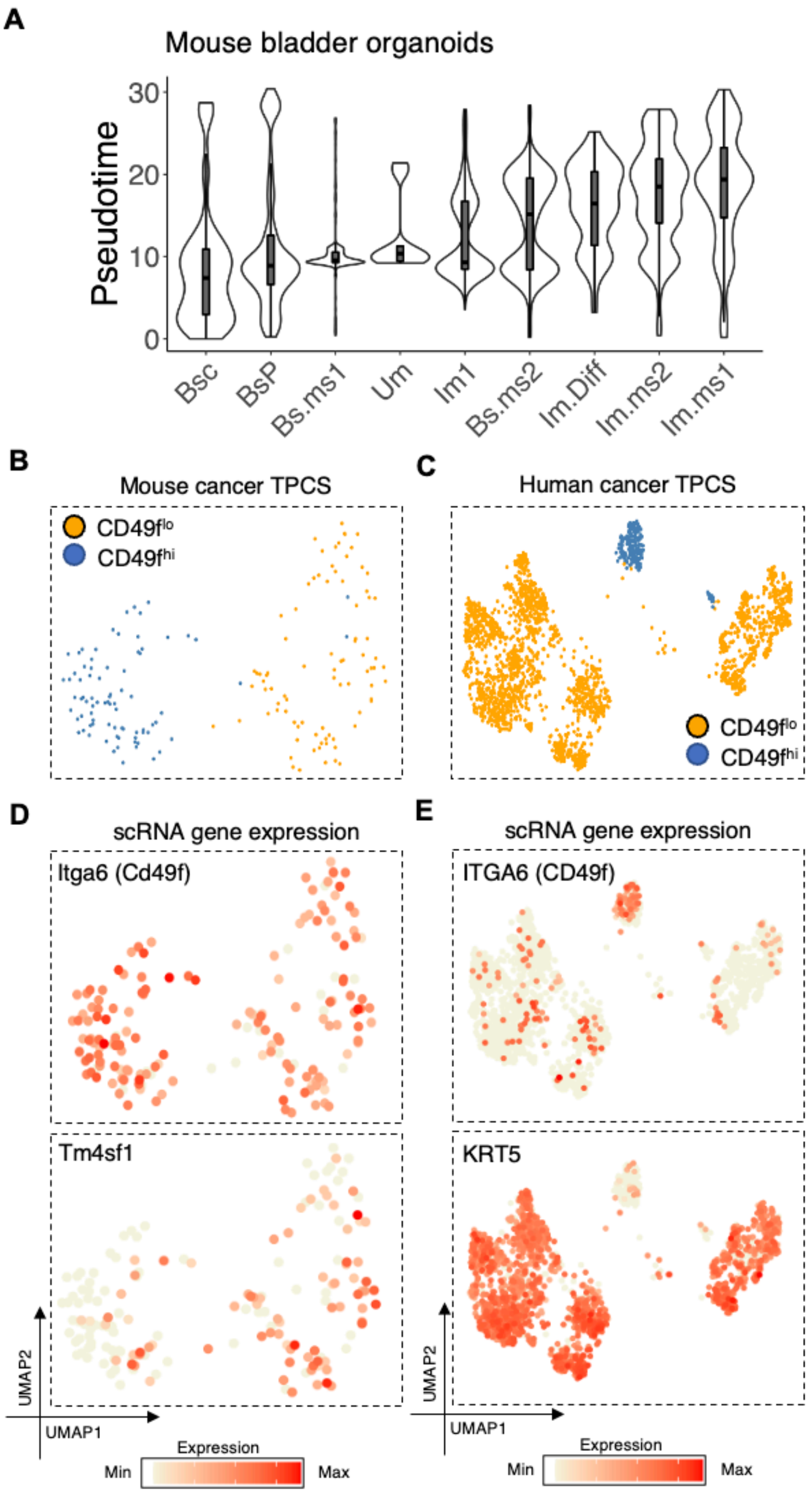

Figure S11. Developmental pseudotime of mouse urothelium cells, related to Figure 5.

(A) Developmental pseudotime is inferred by slingshot for mouse epithelial cells in the normal bladder and organoid dataset. Bsc: basal stem cell; BsP: basal intermediate progenitor cell; Bs.ms1, Bs.ms2: basal cells; Um: umbrella cell; Im1, Im.ms1, Im.ms2: intermediate cells; Im.Diff: differentiating intermediate cells. **(B)** UMAP projection of mouse OHBBN induced bladder cancer TPCS cells colored by $Cd49f^{high}$ (blue) and $Cd49f^{low}$ (orange). **(C)** UMAP projection of human bladder cancer TPCS cells colored by $CD49f^{high}$ (blue) and $CD49f^{low}$ (orange). **(D)** scRNA expression of Itga6 (Cd49f) and Tm4sf1 on UMAP projection in (B). **(E)** scRNA expression of ITGA6 (CD49f) and KRT5 on UMAP projection in (C).

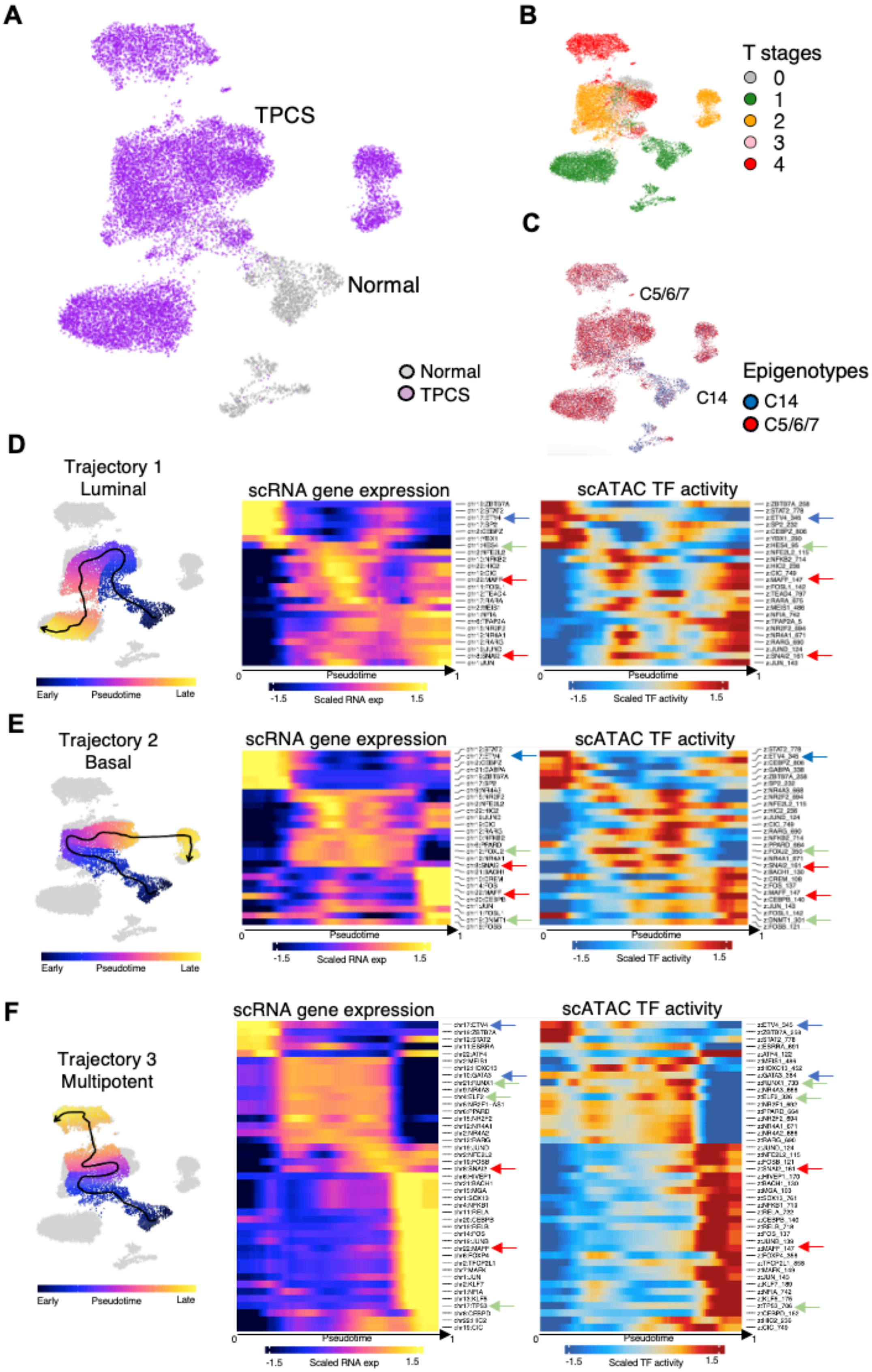
A
TPCS
Normal
Normal
TPCS
B
T stages
0
1
2
3
4
C
C5/6/7
C14
Epigenotypes
C14
C5/6/7
D
Trajectory 1
Luminal
Early
Pseudotime
Late
scRNA gene expression
Pseudotime
-1.5
Scaled RNA exp
1.5
scATAC TF activity
Pseudotime
-1.5
Scaled TF activity
1.5
E
Trajectory 2
Basal
Early
Pseudotime
Late
scRNA gene expression
Pseudotime
-1.5
Scaled RNA exp
1.5
scATAC TF activity
Pseudotime
-1.5
Scaled TF activity
1.5
F
Trajectory 3
Multipotent
Early
Pseudotime
Late
scRNA gene expression
Pseudotime
-1.5
Scaled RNA exp
1.5
scATAC TF activity
Pseudotime
-1.5
Scaled TF activity
1.5

Figure S12. Joint scATAC/scRNA analysis of the developmental trajectory from BsP towards TPCS, related to Figure 6.

(A) UMAP projection of scATAC normal (gray) or TPCS (purple) cells. **(B)** Single cells colored by clnical stage of their donor. T0: DCD healthy donor. **(C)** Single cells colored by their epigenotypes. C14: normal urothelium epigenotype; C5/6/7: TPCS epigenotype. **(D-F, left)** Developmental trajectory on UMAP. Single cells belonging to the trajectory are colored by their respective developmental pseudotime (early-to-late: purple to gold). (**D-F, Middle**) integrated scRNA transcription factor gene expression profile across the developmental trajectory. **(D-F, right)** integrated scATAC transcription factor activity profile, of the similar transcription factors as in middle panel, across the developmental trajectory. On the middle and right panels, early developmental stage-specific transcription factors are labelled by blue arrow, late developmental stage-specific transcription factors by red arrow, and lineage-specific transcription factors by green arrow. **(D)** Developmental trajectory 1, towards a GATA3^{high}, luminal-lineage priming TPCS cluster. **(E)** Developmental trajectory 2, towards a ETV4^{high}, basal-lineage priming TPCS cluster. **(F)** Developmental trajectory 3, towards a multilineage-potent TPCS cluster.

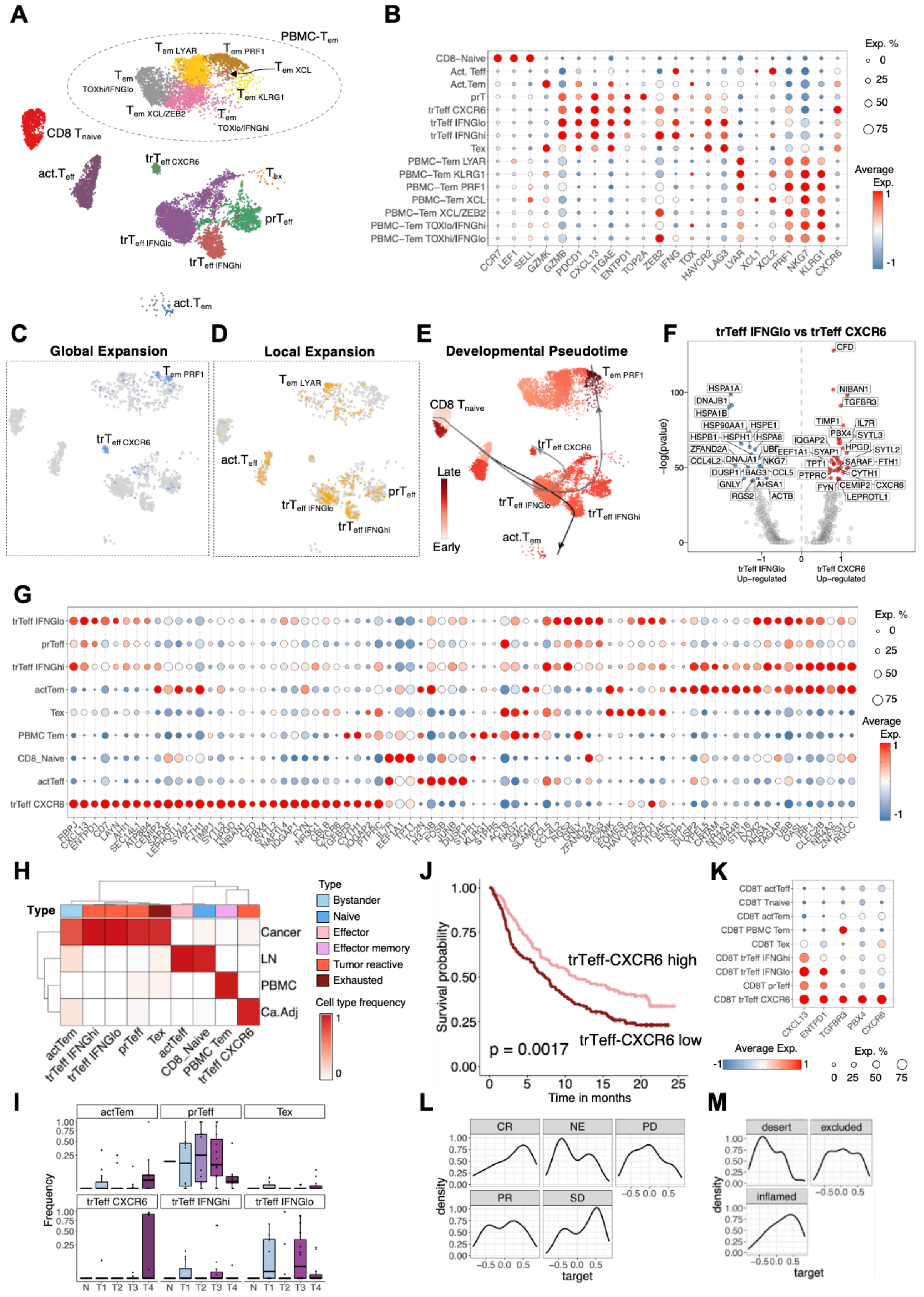

Figure S13. CD8$^+$ T cells in BLCA, related to Figure 7.

(A) UMAP projection of CD8$^+$ T scRNA cells. **(B)** Differential expression of marker genes among the CD8$^+$ T cell clusters in (A). **(C-D)** Single T cells of globally-expanded TCR clones (blue, left) or locally-expanded TCR clones (orange, right). **(E)** Developmental trajectory (arrows) and single cell pseudotime of the CD8$^+$ T cells. **(F)** Differential gene expression between trT$_{eff}$-IFNG^{lo} and trT$_{eff}$-CXCR6 clusters. **(G)** Detailed differential expression of genes in naive T progenitors and tumor-reactive, experienced T$_{eff}$ cells. **(H)** Cell type prevalence of each CD8$^+$ T cell types in sampled tissue. **(I)** Relative cell frequency in donors of different clinical stages. **(J)** Survival probability in IMvigor210 cohort of patients, stratified by trT$_{eff}$-CXCR6 signature gene (TGFBR3, PBX4, CXCR6) expression. **(K)** trT$_{eff}$-CXCR6 gene signature is specific for trT$_{eff}$-CXCR6 cell type. **(L)** Distribution of trT$_{eff}$-CXCR6 gene signature expression (x axis) in patients stratified by immunocheckpoint-inhibitor therapy treatment response. **(M)** Distribution of trT$_{eff}$-CXCR6 gene signature expression (x axis) in patients stratified by histology-classified immune infiltration phenotype.

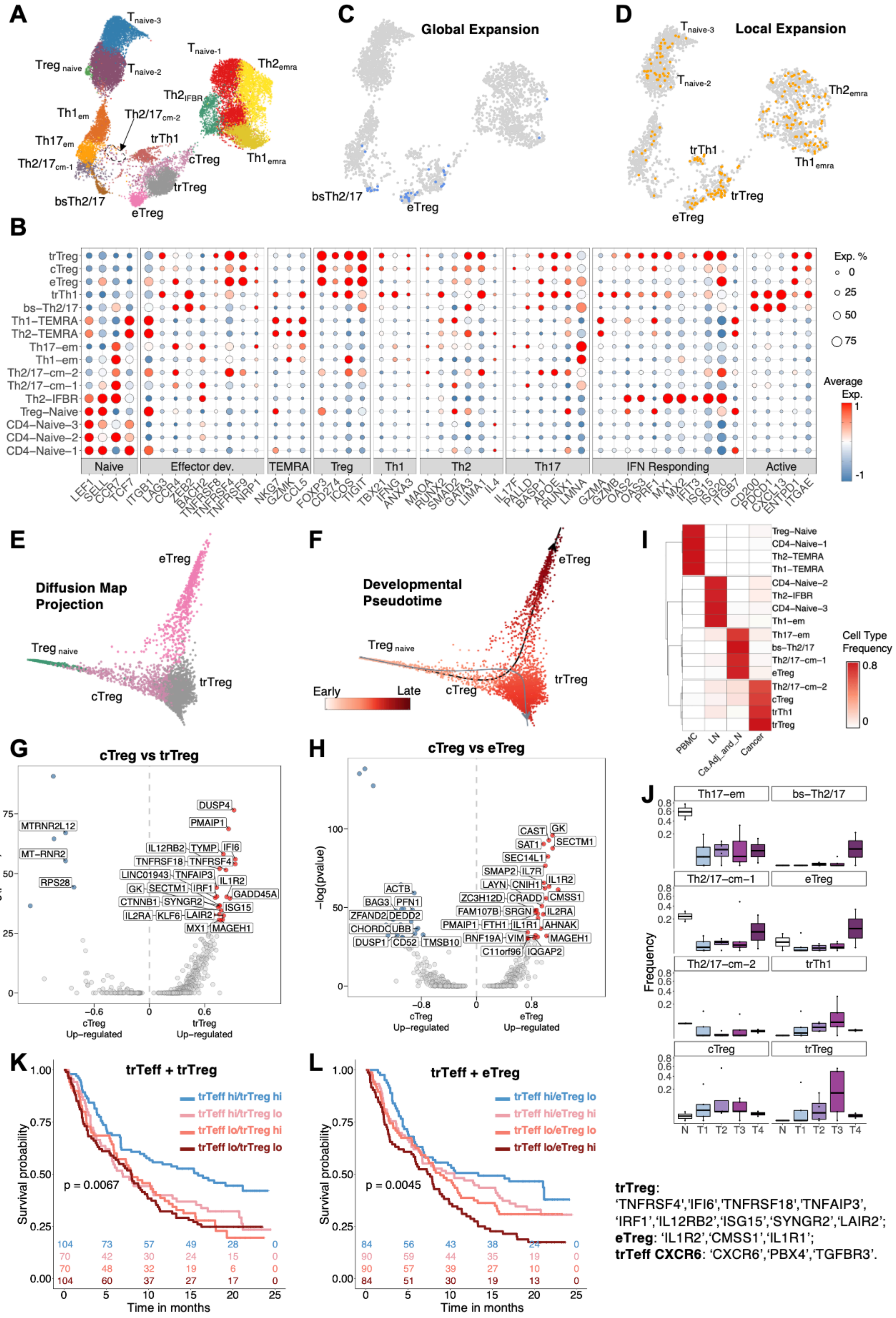
A
Treg naive
Th1em
Th17em
Th2/17cm-1
bsTh2/17
eTreg
trTreg
cTreg
trTh1
Th2/17cm-2
Th2IFBR
Tnaive-1
Tnaive-2
Tnaive-3
Th2emra
Th1emra
C
Global Expansion
bsTh2/17
eTreg
D
Local Expansion
Tnaive-3
Tnaive-2
Th2emra
trTh1
trTreg
Th1emra
eTreg
B
trTreg
cTreg
eTreg
trTh1
bs-Th2/17
Th1-TEMRA
Th2-TEMRA
Th17-em
Th1-em
Th2/17-cm-2
Th2/17-cm-1
Th2-IFBR
Treg-Naive
CD4-Naive-3
CD4-Naive-2
CD4-Naive-1
Naive
Effector dev.
TEMRA
Treg
Th1
Th2
Th17
IFN Responding
Active
LEF1 SELL CCR7 TCF7 ITGB1 LAG3 CCR4 ZEB2 BACH2 TNFRSF8 TNFRSF4 TNFRSF9 NRP1 NKG7 GZMK CCL5 FOXP3 CD274 ICOS TIGIT TBX21 IFNG ANXA3 MAOA RUNX2 SMAD2 GATA3 LIMA1 IL4 IL17F PALLD BASP1 APOE RUNX1 LMNA GZMA GZMB OAS2 OAS3 PRF1 MX1 MX2 IFIT3 ISG15 ISG20 ITGB7 CD200 PDCD1 CXCL13 ENTPD1 ITGAE
Exp. %
0
25
50
75
Average Exp.
1
-1
E
Diffusion Map Projection
eTreg
Treg naive
cTreg
trTreg
F
Developmental Pseudotime
eTreg
Treg naive
Early
Late
cTreg
trTreg
I
Treg-Naive
CD4-Naive-1
Th2-TEMRA
Th1-TEMRA
CD4-Naive-2
Th2-IFBR
CD4-Naive-3
Th1-em
Th17-em
bs-Th2/17
Th2/17-cm-1
eTreg
Th2/17-cm-2
cTreg
trTh1
trTreg
Cell Type Frequency
0.8
0
PBMC
LN
Ca.Adj_and_N
Cancer
G
cTreg vs trTreg
-log(pvalue)
MTRNR2L12
MT-RNR2
RPS28
DUSP4
PMAIP1
IL12RB2 TYMP IFI6
TNFRSF18 TNFRSF4
LINC01943 TNFAIP3
GK SECTM1 IRF1 IL1R2
CTNNB1 SYNGR2 GADD45A
IL2RA KLF6 LAIR2 ISG15
MX1 MAGEH1
-0.6
0
0.6
cTreg Up-regulated
trTreg Up-regulated
H
cTreg vs eTreg
-log(pvalue)
CAST GK
SAT1 SECTM1
SEC14L1
SMAP2 IL7R
LAYN CNIH1 IL1R2
ZC3H12D CRADD CMSS1
FAM107B SRGN IL2RA
PMAIP1 FTH1 IL1R1 AHNAK
RNF19A VIM MAGEH1
C11orf96 IQGAP2
ACTB
BAG3 PFN1
ZFAND2 DEDD2
CHORDC1 UBB
DUSP1 CD52 TMSB10
-0.8
0
0.8
cTreg Up-regulated
eTreg Up-regulated
J
Th17-em
bs-Th2/17
Th2/17-cm-1
eTreg
Th2/17-cm-2
trTh1
cTreg
trTreg
Frequency
N T1 T2 T3 T4
K
trTeff + trTreg
trTeff hi/trTreg hi
trTeff hi/trTreg lo
trTeff lo/trTreg hi
trTeff lo/trTreg lo
p = 0.0067
Survival probability
104 73 57 49 28 0
70 42 30 24 15 0
70 48 32 19 6 0
104 60 37 27 17 0
Time in months
L
trTeff + eTreg
trTeff hi/eTreg lo
trTeff hi/eTreg hi
trTeff lo/eTreg lo
trTeff lo/eTreg hi
p = 0.0045
Survival probability
84 56 43 38 24 0
90 59 44 35 19 0
90 57 39 27 10 0
84 51 30 19 13 0
Time in months
trTreg: 'TNFRSF4','IFI6','TNFRSF18','TNFAIP3', 'IRF1','IL12RB2','ISG15','SYNGR2','LAIR2';
eTreg: 'IL1R2','CMSS1','IL1R1';
trTeff CXCR6: 'CXCR6','PBX4','TGFBR3'.

Figure S14. $CD4^+$ T cells in BLCA, related to Figure 7.

(A) UMAP projection of $CD4^+$ T scRNA cells. **(B)** Differential expression of marker genes among the $CD4^+$ T cell clusters. Known $CD4^+$ T cell marker gene annotations are labeled by the bottom strips. **(C)** Single T cells of globally-expanded TCR clones (blue) or **(D)** locally-expanded TCR clones (orange). **(E)** Diffusion map of effector regulatory T cells (eT_{reg} and trT_{reg}) developing from naive T_{reg}. **(F)** Developmental trajectory (arrows) from naive T_{reg} through cT_{reg} towards trT_{reg} or eT_{reg}. **(G)** Differential gene expression between cT_{reg} and trT_{reg}. **(H)** Differential gene expression between cT_{reg} and eT_{reg}. **(I)** Cell type prevalence of each $CD4^+$ T cell types in sampled tissue. **(J)** Relative cell frequency in donors of different clinical stages. **(K)** Survival probability in IMvigor210 cohort of patients, stratified by trT_{eff}-CXCR6 signature gene expression and trT_{reg} signature gene expression. **(L)** Survival probability in IMvigor210 cohort of patients, stratified by trT_{eff}-CXCR6 signature gene expression and eT_{reg} signature gene expression. Signature genes are shown on the right.

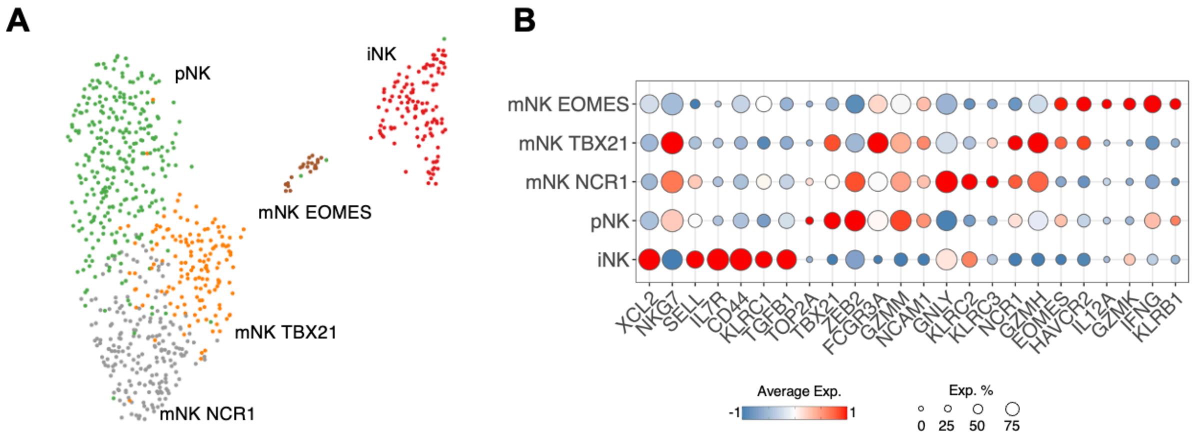

Figure S15. NK cells in BLCA.

(A) UMAP projection of NK scRNA cells. **(B)** Differential expression of marker genes among the NK cell clusters.

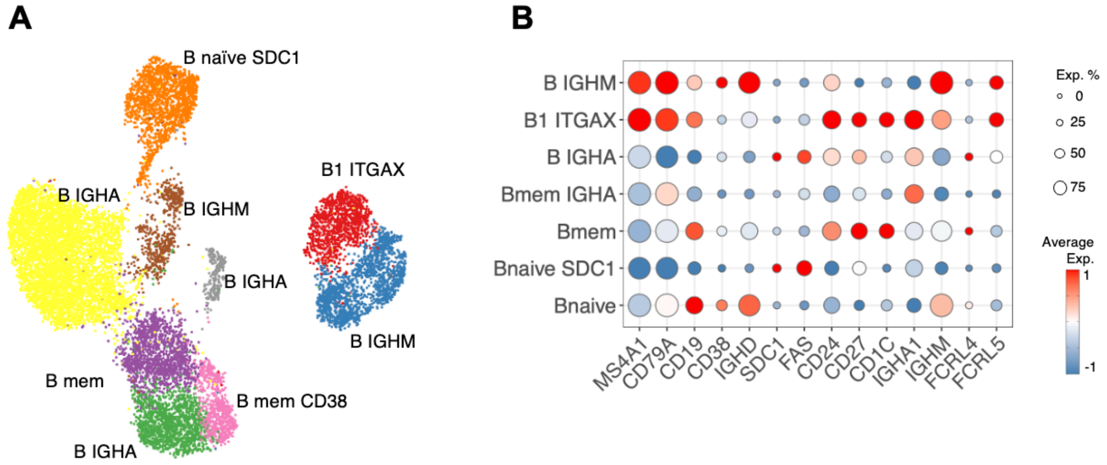

Figure S16. B cells in BLCA.

(A) UMAP projection of B scRNA cells. **(B)** Differential expression of marker genes among the B cell clusters.

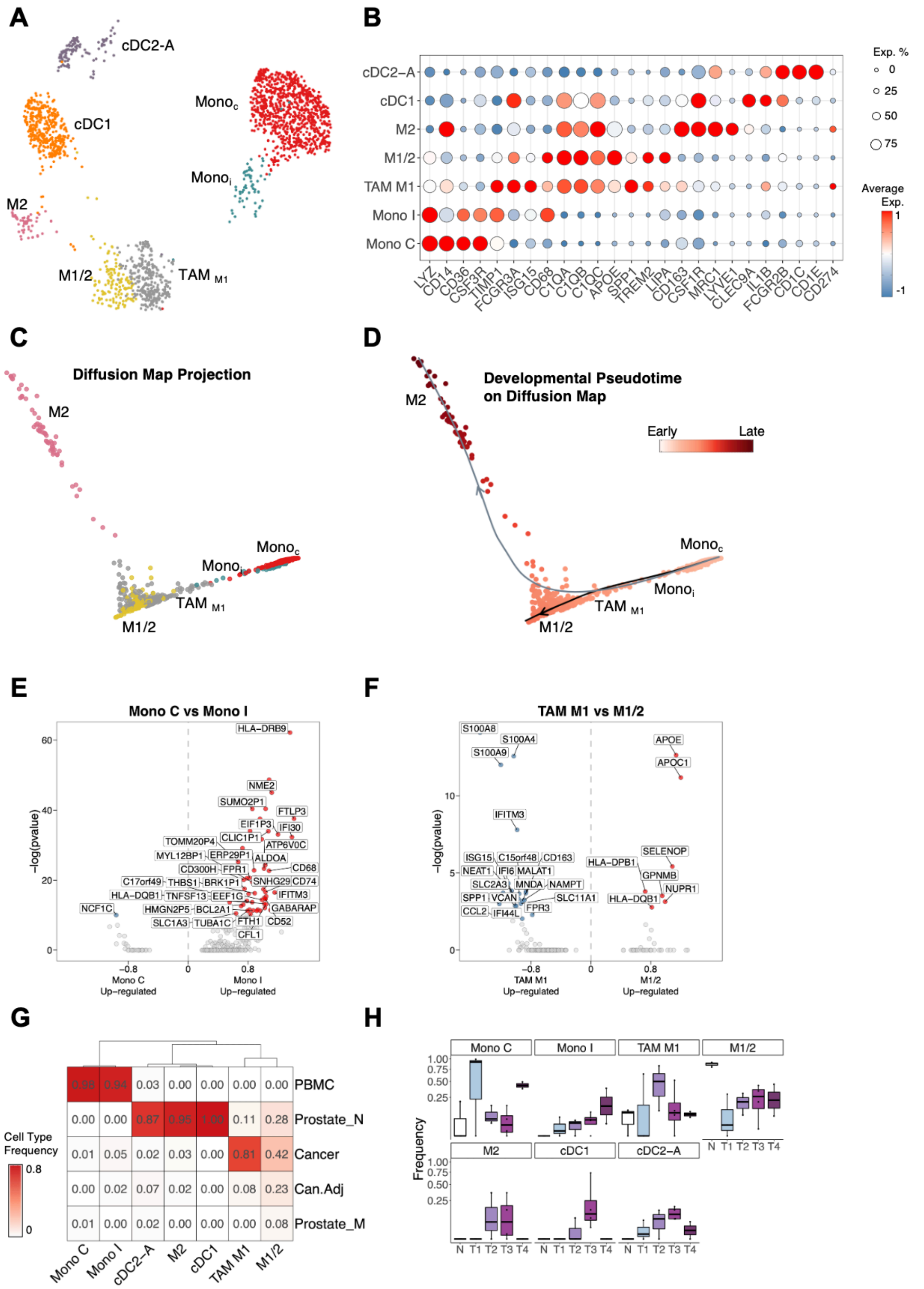

Figure S17. Myeloid-derived cells in BLCA.

(A) UMAP projection of myeloid-derived scRNA cells including monocytes, dendritic cells, and macrophages. **(B)** Differential expression of marker genes among the myeloid cell clusters. **(C)** Diffusion map projection of the monocyte-macrophage lineage. **(D)** Developmental trajectory and pseudotime of the monocyte-macrophage lineage. **(E)** Differential gene expression between classical (Mono C) and intermediate monocyte (Mono I). **(F)** Differential gene expression between tumor-associated macrophage M1 (TAM M1) and non-tumor-associated M1/M2 macrophage (M1/2). **(G)** Distribution of myeloid cells in sampled tissue. **(H)** Relative cell frequency in donors of different clinical stages.

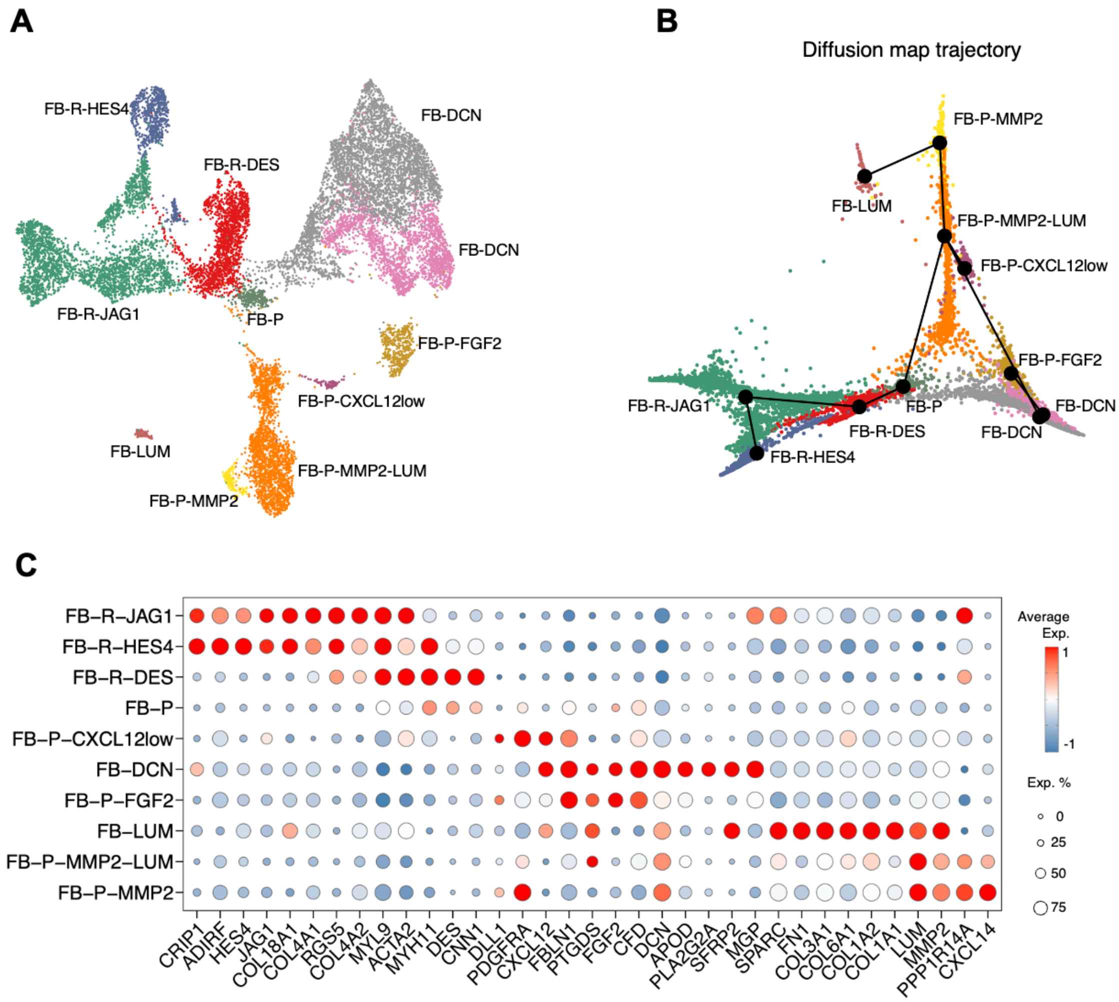

Figure S18. Analysis of fibroblast cells in scRNA dataset, related with Supplementary Notes. (A) Classification of scRNA fibroblast (FB) cells. Cell types are denoted by their specific marker gene expression. R: RGS5-positive; P: PDGFRA-positive. **(B)** Diffusion map inferred trajectory of fibroblasts. **(C)** Differential expression of marker genes in each fibroblast cell cluster.

Description of Supplementary Datasets 1-9

Supplementary Dataset 1. Human biospecimen and sequencing details.

Sheet "Notes" describes the abbreviation of diseases and treatments used in sheets "Donors" and "Experiment". Sheet "Donors" describes the information for each donor involved in this study, with the following information: individual name ("Individual_name"), gender, disease information ("Disease_information"), disease stage in TNM staging format ("Disease_stage"), Treatment (chemotherapy, resection, or TURBT), number of sample used in the study ("Nsamples"), list of sample ID ("Sample_list"), list of tissues ("Tissue_list") and list of assays ("Experiment_type"). Sheet "Experiment" shows the information associated with each experiment, including donor-specific information, sample-specific information, as well as the associated specific assay type ("Experiment_type") and tissue type ("Tissue").

Supplementary Dataset 2. **Autosomal and X copy number segments output by CNVkit.**

Associated case-control pairs ("Case_sample", "Control_sample") are shown. The reference genome is hg19/b37.

Supplementary Dataset 3. **SNV mutations of each sample.**

The reference genome is hg19/b37. Annotation is formatted by VEP. The variant ("Variant_hg19") and associated gene ("Gene"), cDNA-level change ("HGVSc"), protein-level change ("HGVSp"), mutation type ("Consequence") are shown. "Mutation_class" describes whether the mutation is germline or somatic. In cases without a proper normal control, the mutation class is set to be

"unknown". MCAP prediction and population frequencies (gnomAD_EAS_AF and gnomAD_AF) are shown.

Supplementary Dataset 4. scRNA cell annotations.

QC-passed scRNA cell name list with its cell type annotation.

Supplementary Dataset 5. scRNA cell type markers.

Differentially expressed genes between different cell types as output by Seurat::FindAllMarkers.

Supplementary Dataset 6. **MIBC associated DMR loci.**

Reference genome is hg38. Differentially methylated region (DMR) location ("Chr_name", "Start", "End") and class are shown. "Class" describes whether the DMR region is hypermethylated or hypomethylated in MIBC compared to NMIBC.

Supplementary Dataset 7. scATAC marker peaks for epithelial cell type.

Reference genome is hg38. Differentially accessible peaks among epithelia subtypes identified by ArchR::getMarkerFeatures. "Origin" describes the specific cell-type-of-origin for this peak.

Supplementary Dataset 8. **scATAC of non hematopoitic lineage epigenotype cluster annotation.**

scATAC with its matched gross cell type ("Sample_type"), cell cluster according to global chromatin accessibility ("Seurat_clusters_Group"), cell cluster according to CNV-free DMR chromatin accessibility ("Seurat_clusters_evolution_Group"), matched scRNA cell

("Predicted_Cell_RNA") and matched RNA cluster ("Seurat_clusters_epithelial_final_RNA_Group").

Supplementary Dataset 9. Details of cisTarget TPCS NMF.

Motifs and their annotated TF among enriched gene sets in TPCS NMF6/11/16 in standard format output by Rcistarget.

Supplementary Dataset 10. Glossary of abbreviations.

A detailed list of gene symbols, full names and brief descriptions of gene functions.
